# Effectiveness of group-based psycho-education on preventing postpartum depression among pregnant women by primary healthcare provider in primary healthcare institution: a cluster-randomized controlled trial

**DOI:** 10.3389/fpsyt.2024.1433942

**Published:** 2024-09-10

**Authors:** Marta Tessema, Muluemebet Abera, Zewdie Birhanu

**Affiliations:** ^1^ School of Midwifery, Institute of Health, Jimma University, Jimma, Ethiopia; ^2^ Department of Population and Family Health, Faculty of Public Health, Jimma, Ethiopia; ^3^ Department of Health, Behavior and Society, Faculty of Public Health, Jimma, Ethiopia

**Keywords:** postpartum depression, psycho-education, effectiveness, randomized controlled trial, primary health care

## Abstract

**Background:**

In Ethiopia, one in five mothers suffers from postpartum depression, which needs to be prevented through interventions. According to the World Health Organization, maternal healthcare providers have a unique opportunity to provide psychosocial interventions to prevent the damaging effects of perinatal depression. Hence, this study assessed the effectiveness of prenatal group-based psycho-education in preventing postpartum depression (PPD) in primary healthcare units.

**Methods:**

We conducted a two-arm cluster-randomized controlled trial, enrolling 550 pregnant women at 12–20 weeks of gestation with a normal score (0–4) and a mild score (5–9) on the Patient Health Questionnaire-9 (PHQ-9). The study utilized simple randomization techniques to assign clusters between arms in a 1:1 ratio. The data was collected through face-to-face interviews conducted at 12-20 weeks of gestation and 6 weeks postpartum. The intervention group received usual care plus five prenatal group-based psycho-education (PGBPE) classes, while the control group received only usual care. The PPD status between arms was compared using the chi-square test of association. A mixed-effects multilevel logistic regression model was also used to examine the predictors of the outcome variables.

**Results:**

The overall response rate at the end line was 92.9%. Thus, compared to that in controls, the PPD in the intervention clusters was considerably lower (20 (7.6%) *vs*. 74 (28.9%)), P = 0.001)/65% (AOR = 0.35, 95% CI = 0.13–0.99), although no difference was detected at baseline. Social support (AOR = 0.04, 95% CI = 0.01–0.15), partner emotional support (AOR = 0.24, 95% CI = 0.12–0.51), PPD literacy (AOR = 0.25, 95% CI = 0.11–0.62), and self-esteem (AOR = 0.22, 95% CI = 0.11–0.47) were more likely to protect mothers from PPD. On the contrary, domestic work (AOR = 9.75, 95% CI = 3.37–28.16), neonates with complications (AOR = 5.79, 95% CI = 2.04–16.45), and unhealthy coping (AOR = 2.39, 95% CI = 1.06–5.42) exposed mothers to PPD.

**Conclusion:**

The implementation of a PGBPE in primary healthcare units (PHCUs) was effective at preventing PPD. Therefore, this intervention method has to be promoted and used in PHCUs to prevent PPD.

**Clinical Trial Registration:**

[Pan African Clinical Trial Registry], identifier [PACTR 202203616584913].

## Introduction

Women are at high risk of depression, with a lifetime risk of 10–25% (1). This heightened vulnerability to depression begins in puberty and lasts until menopause. It goes up during the perinatal period due to both biological and psychosocial factors related to pregnancy and childbirth (1, 2). Perinatal depression is a term used for major depression episodes during pregnancy and/or a year after the birth or adoption of a baby (3, 4). The global prevalence of perinatal depression (PND) ranges from 15 to 65% (5). Its pooled prevalence in low- and middle-income nations was 34% and 22.7%, respectively (6). In East Africa, its range is estimated to be up to 24% (7). In Ethiopia, the pooled prevalence of perinatal depression is 25.8% (8). Furthermore, antenatal and postnatal depression account for (9) and 21.9% (10), respectively.

Postpartum depression impairs and disturbs a woman's function, health-promoting behavior, and interpersonal relationships, which can cause poor birth outcomes, couple dysfunction, and child abuse (11). Furthermore, it is related to the loose mother-child attachment and the mother's unresponsiveness to the child's needs or her failure to recognize the baby's cue, which would have a potential impact on the baby's development (12). Among others, early cessation of breastfeeding (13) and receiving less preventive care like vaccinations and a balanced diet can be the consequence (14). As a result, the healthcare system, their families, and society would bear a significant burden.

In the past, effective antidepressant and psychotherapeutic interventions were applied to treat PND, but PND has remained an inadequately treated medical problem, particularly in low- and middle-income countries. In the literature, several reasons have been mentioned, such as the safety of antidepressant drugs during pregnancy and lactation (15), and the general societal stigma concerning mental healthcare (16). Moreover, compliance with psychotherapeutic interventions being West-centric and indifferent to various cultural and religious beliefs (17), and a shortage of specialized mental health professionals have also been identified as causes (18). Pertinent to this, the Program to Reduce the Treatment Gap for Mental Disorders recommended that PND treatments and training programs in low- and middle-income countries be responsive to the local culture. In addition, they must incorporate a public health approach and meet the diverse needs of mothers (17, 19).

Moreover, Studies found that three key hypothalamic-pituitary-adrenal axis hormones—corticotropin-releasing hormone, adrenocorticotropic hormone, cortisol, and reproductive hormones (progesterone and oxytocin)—were significant predictors of PPD. Also, thyroid autoimmunity-related thyroid dysfunction, higher levels of proinflammatory cytokines, and lower levels of zinc, C-reactive protein, and vitamin D in the blood are all strong predictors of postpartum depression (20). And psychosocial (like poor social support) (2, 21) were risk factors for PPD. However, it has also been highlighted that biological factors alone are not sufficient to manifest the disorders; rather, they could require psychosocial stressors (2, 21). Based on this evidence currently to prevent PND almost all interventions are focused on psychosocial stressors.

Prevention strategies can be in all three levels. Mostly, in low- and middle-income countries the strategies focused on secondary and tertiary levels of prevention (22). Both community- and facility-level interventions that can be applied by primary healthcare practitioners such as psychotherapy (CBT,IPT),and psycho-education which includes social support, couple-focused education, income generation programs, task sharing, mHealth, and other interventions related with child care were tried in these countries (22–26). However, most of the studies did not identify the most common risk factors in the study population that they were seeking to address. Furthermore, evidence shows primary prevention strategies, which are offered for non-depressed pregnant women and are the most cost-effective, less stigmatizing, and more likely to be used by participants, were more effective (22). So, this study tried to fill this gap and targeted poor social support and PND literacy (27, 28), which are the most important psychosocial risk factors in the study area. Furthermore, to increase women's PPD literacy and fulfill the social support needs of the women, prenatal group-based psycho-education is considered a viable method of intervention for women and their families (spouses).

Psycho-education is a practice that provides the patient and families knowledge about various facets of the illness and its prevention so that they can work together with professionals for a better overall outcome (29). The idea is that the more informed the care recipients and informal caregivers are about PND and the necessity of social support, the greater assistance the mother receives (29). Moreover, social support, which is the cornerstone of psycho-education, functions as a buffer against the effects of psychiatric vulnerability and life stressors. Research also indicates that extending social support to women who are childbearing is a crucial intervention in preventing PND (26, 30). Furthermore, group-based psycho-education typically assists clients, where the majority of common questions raised by participants are addressed predominantly through discussion and experience sharing within the groups, which helps the pregnant women feel like they are not alone and more confident in the group's support (21, 31). Several fully powered randomized trials on maternal healthcare services have shown the effectiveness of this intervention in preventing postpartum depression (PPD) among low-income women in the first 6 months after childbirth (23, 32–36). It is flexible and easy to implement, and it also helps manage attendance barriers (21, 29). Furthermore, the World Health Organization (WHO) recommended it for busy, low- and middle-income country health facilities by non-specialized healthcare providers in non-specialized healthcare facilities (18).

Regarding the timing of intervention, evidence shows that almost all stressors are common for both antenatal and postpartum depression (2, 21). They increase over the third trimester and in the immediate postpartum periods (6). Furthermore, the Diagnostic and Statistical Manual-5 accredited that the peripartum onset specifier and 40% of postpartum major depressive episodes begin during pregnancy (14). In line with this, the WHO, the American College of Obstetricians and Gynecologists, and Postpartum Support International recommended that universal screening and psychosocial support during the antenatal period are crucially vital (37). So, to effectively mediate both biological and psychosocial risk factors and use prevention strategies to prevent PPD, psychosocial interventions must be started early or around mid-pregnancy (12–20 weeks) when the woman is in stable emotional status (normal and mild depressive symptoms) when she clearly understands PPD and prepares herself for healthy coping (38, 39).

In line with the aforementioned discussions, the present study aimed to assess the effectiveness of prenatal group-based psycho-education in preventing postpartum depression in the primary healthcare unit. To this effect, a cluster-randomized control trial was applied. The main reason for applying a cluster-randomized control trial was that a component of this study had already been in use at the facility level so that the defect of information contamination could be controlled.

## Materials and methods

### Study design

The findings were reported per the CONSORT reporting criteria (See **Supplementary Table 1**). A cluster-randomized controlled trial with parallel-group, single-blind, two-arm intervention, and a 1:1 allocation ratio was performed. This method was used to compare prenatal group-based psycho-education interventions with the usual, standard care system as a control arm. Moreover, since the researchers' goal was to implement this intervention on the entire maternal healthcare service in the facility, health center clusters were taken as the units of randomization.

### Source population

All pregnant women who fulfilled the study's eligibility criteria and attended prenatal care in a primary health care facility in the study area.

### Study population

All systematically selected pregnant women who fulfilled the study's eligibility criteria and attended prenatal care in a primary health care facility in the study area.

### Eligibility criteria

Health centers that were provided with female midwives and nurses who had ≥6 months of experience in maternal healthcare services and were familiar with the local languages and culture were included in the study. Accordingly, out of 122 health centers in the zone, 32 non-adjusted health centers were included. Thus, the study participants were pregnant women who were 12–20 weeks of gestation and had a normal score (0–4) or mild score (5–9) on the scale of depression status measured by PHQ-9. The remaining mothers with moderate-severe depression (PHQ-9 ≥ 10) in both groups were referred to a nearby hospital for further evaluation and treatment by distinct clinicians (psychiatry nurses).

### Exclusion criteria

Mothers who had been on treatment for previous mental illnesses had hearing disabilities, or were severely ill were also excluded from the study.

### Study period and setting

The trial was conducted from 28/3/2022 to 01/12/2022 in primary healthcare facilities (health centers) found in the Jimma Zone, part of the Oromia Regional State, Southwestern Ethiopia. According to the 2007 census, in this zone, the total population was 3,486,155, of which the number of females was 1,735,628 (40). Structurally, the population is patrilineal, and the patrilocal setting is frequently characterized by unequal gender norms and limited female decision-making capacity (41). In the zone, there are 6 primary hospitals with a distribution of 1 general hospital, 1 referral hospital, 122 health centers, and 548 health posts. Women typically receive maternal healthcare services from primary healthcare units (primary hospitals, health centers, and health posts), which are primarily served by midwives and nurses. In the zone, there were no maternal mental health services or interventions in any maternal healthcare services in the facility (27).

### Randomization and sampling procedure

Following the completion of cluster recruitment, SPSS-generated random sequences were used to assign clusters to the intervention and control arms at a 1:1 allocation ratio. A person, who was blinded to study groups and did not participate in this study, randomized the clusters and allocated them into two arms, since the goal of the intervention was to improve the use of evidence in the entire maternal healthcare service in the facility, the primary healthcare unit (health center) was taken as the unit of randomization. To this effect, by taking three consecutive monthly reports from each facility, the average monthly load of antenatal care clients within the specified gestational age (12–20 weeks) was estimated. Accordingly, using a systematic sampling technique, every two pregnant women were selected until the required sample size was obtained. If the selected mothers failed to fulfill the eligibility criteria, the next mothers were included in the study. The identification and consent of mothers for the baseline survey were performed before the randomization procedure was performed, and this helped the researchers minimize identification biases.

### Blinding

This study was single-blind; only the outcome assessors were concealed, were not informed about the allocation and objective of the study, did not live in any of the clusters, and were not trial implementers. To increase the experience sharing and active involvement of the mothers in the intervention, the study participants were not concealed; they were well aware of the objective of the study. It was also challenging to conceal providers (those who delivered interventions) because the intervention was education; thus, they knew what type of health education and counseling services were given to mothers in the prenatal period in all areas of the region.

### Intervention activities for the intervention arm

To implement group-based psycho-education at both the provider level (facility level) and the mother level (individual level), ample training was given to the providers. Facility-level training was needed because the intervention was planned to be delivered by maternal healthcare providers, midwives, and nurses. Thus, the study was delivered in groups, and in each group, 8–10 pregnant women participated. In total, 5 prenatal group sessions lasting from 60 to 90 minutes were administered every week for a total of 5 weeks to all intervention groups. In the intervention, baby blues and postpartum depression, symptoms of PPD and when to recognize them, prevention methods, and the development of social support were included. Moreover, in the last week, mothers were asked to bring their partner or family member. Then, discussions were held on common psychosocial problems that can occur during pregnancy and childbirth, including the importance of family support in managing stress and preventing PPD. A detailed description of the intervention is presented in **Table 1**.

**Table 1:** Summary of intervention activities.

Session	Key messages
Baby blues and PPD	The participants became familiar with each other and the structure of the sessions. In the continuation of the same session. Participants were asked about perinatal depression, and discussions were conducted on how it is seen in our community. The presence of stigma related to PPD and its beliefs were discussed. Finally, the clinical definition of PPD, the difference between baby blues and PPD, including risk factors and causes, the potential impact on the mother, pregnancy outcome, and long-term effects on the baby and family as a whole, were discussed by the midwives.
Symptoms of PPD and when to recognize it	A recap was done and continuously participant was asked about symptoms of perinatal depression and discussions were conducted in this regard. Finally, the clinical symptoms of PPD and when to recognize them were discussed. Mothers were asked to check the presence and absence of those symptoms or feelings in them and how long the feeling stayed with them (it is done to make sure the mothers identify symptoms of PPD and know when to diagnose PPD). Finally, midwives were discussed, as PPD is a preventable and treatable problem with self-care activities and the availability of professional help (counselling and medication).
Prevention methods	Participants were asked about prevention and treatment methods for depression in the community and discussions were conducted in this regard. Then, the midwives discussed the prevention method based on common cause/risk factors that are commonly present around pregnancy and childbirth (such as social support, economic problems, conflict, and workload). Participants were asked to think about their current emotional feelings and what was the cause of those feelings (for the exact cause of their stress). In addition, a discussion was conducted about how they respond to that cause of stress. Finally, midwives discussed the healthy and unhealthy ways of coping with stress.
Social support for the prevention of PPD	Continued prevention methods and healthy coping, including the importance of social support during pregnancy and once the baby is born and its benefits in managing stress, and forms of social support and how to get social support. Group discussion and experience sharing were conducted on how to get social support, including how to involve partners in child care and household chores positively. Information was provided about community groups such as the 1-5 network and a women's forum; they encouraged joining the group.
Discussions with partners or any person the mother wants	Discussions with partners or family members were done about PPD and its risk factors, the potential impact on the mother, pregnancy outcome, and long-term effects on the baby and family as a whole. The need for family help for healthy coping and prevention of PPD was discussed. Finally, the areas of support (emotional (expressions of empathy, love, trust, and care), instrumental (tangible aid), informational (advice, and suggestions), and appraisal were discussed by midwives.

### Control arm

Participants in the control arm received only the existing routine care (focused antenatal care). Thus, routine care included no assessment, management, or prevention programs related to PND (maternal mental health).

### Description of the interventions according to the TIDieR checklist

#### Brief name

Prenatal group-based psycho-education intervention

#### Rationale, theory, or goal of the elements essential to the intervention

In line with the aforementioned discussions in the introduction, PND has remained an inadequately treated medical problem, particularly in low- and middle-income countries including Ethiopia. Nevertheless, promising solutions have been identified. For instance, based on the stress process and stress vulnerability models, stressors and mediators play a role in the development of a woman's mental health outcomes. Stressors are considered both biological and psychological risk factors for PPD. However, the presence of psychosocial stressors is necessary for PPD to occur even when a person is biologically vulnerable. Mediators can be elements that assist mothers in managing stressors in a healthy manner and preventing themselves from PND (2, 21). So based on this evidence, even though perinatal women are vulnerable to both sources of stressors, PPD can be prevented by identifying the key mediators and working with them.

In this study, we used social support and PPD literacy as key mediators to prevent PPD. Because, evidence shows poor social support, particularly spousal support (38, 42), and low PPD literacy (27) were the most important risk factors in the study area for PPD. So, the prenatal group-based psycho-education intervention was applied to cover these both factors and prevent PPD. Furthermore, Primary prevention strategies are offered for non-depressed pregnant women. For this purpose, only women with non-depressed and mild depressive symptoms and 12–20 weeks of gestation were included in the study because, at this time, both the psychosocial and biological vulnerability of the mother to stress are very low. When she is in stable emotional status, she can effectively follow interventions and understand the effects of PPD, build positive relationships with people, and prepare for healthy coping. It is the most cost-effective, less stigmatizing, and more likely to be used in non-specialized health facilities and by non-specialized healthcare providers.

Psycho-education focuses on providing knowledge about various aspects of PPD and its prevention to pregnant women, and their families. The idea is that the more informed the care recipients and informal caregivers are about PPD and the necessity of social support, the greater assistance the mother receives (43). Furthermore, the intervention was given in groups, which offered its own set of benefits for the women. The majority of common questions raised by pregnant women were addressed predominantly through discussion and experience sharing within the groups, which helped the pregnant women feel like they were not alone and more confident in the group's support (30, 34).

#### Materials used in the intervention (those provided to participants or used in intervention delivery or training of providers)

The adaptation of the intervention material was carried out in three steps. First, an analysis of the literature review was performed to determine the most common psychosocial risk factors with significant effects on the development of PPD in the study area. In this vein, poor social support, particularly spousal support (28, 42), and poor PPD literacy (27) were identified as common psychosocial problems in the study area. Next, the intervention content and psycho-educational techniques used were derived from different well-validated guidelines, manuals, and handbooks. These sources were prepared for the prevention and management of perinatal depression and postpartum depression prevention programs in low-income women's prenatal clinics (18, 21, 29, 39, 44, 45). Finally, discussions were held with researchers and experts in the area (psychiatrists and psychologists) and with non-specialized healthcare providers (midwives, nurses, and obstetricians) to select the most relevant information that could be incorporated into the intervention. In the end, a complete intervention package explaining the actions to be performed while carrying out this trial was produced for both the provider and the participant pregnant women.

The intervention package given to the provider included relevant aspects of the intervention, such as the antenatal psychosocial assessment tool (PHQ-9), and a procedural guideline for facilitating group psycho-education. All the prenatal group-based psycho-education sessions given to the study participants and procedures for referring women with moderate-severe depression to a nearby hospital were also included. Moreover, methods of monitoring the intervention to decrease the dropout rate of mothers from the intervention and motivating mothers to follow the sessions strictly up to the end-line data collection time were also highlighted for healthcare providers.

Similarly, 5 prenatal group sessions, which included baby blues and postpartum depression, symptoms of PPD and when to recognize it, prevention methods, and the development of social support, were briefed for the participant mothers. Moreover, in the last week, mothers were asked to bring their partner or family member, and then discussions were held on common psychosocial problems that would occur during pregnancy and childbirth and on the importance of family support to manage stress and prevent PPD. A detailed description of the intervention is presented in **Table 1**.

#### Procedures, activities, and/or processes used in the intervention

A log frame was developed with providers and supervisors to pinpoint the participants' objectives and activities. On the first day of the intervention, the participants were introduced to each other and were also informed about the arrangement of the sessions. As the session progressed, group norms were established, group secrets were maintained, and mothers were encouraged to talk openly and share their experiences. A session summary was performed at the end of each session, and the session was recapped before the start of every other session.

Follow-ups were performed strictly to encourage mothers to finish all the sessions. A dropout tracing mechanism was developed; the attendance and address of participants (phone number, kebele, and house number) were also registered. Communication with community health extension workers was also created since health extension workers can easily discern each pregnant woman found in their working kebele. Hence, dropouts were informed by the midwife in charge of that particular area where the target mothers belonged with the help of the health extension worker. Supervision was provided by psychiatrist nurses and principal investigators every week.

#### Description of the expertise, background, and specific training given to intervention providers

Detailed group psycho-education training, including PPD screening, was given to midwives and nurses by experienced psychiatrist nurses (assistant professors) and investigators (MSc in clinical midwifery, a PhD fellow in reproductive health at the population and facility health department, certified for positive family therapy) (See **Supplementary Training Manual 1**).

The intervention was delivered for pregnant mothers by maternal healthcare providers (midwives and/or nurses) who were working at the antenatal care clinic. The participants were recruited from the study sites for resource management and convenience. The participants were recruited based on their sex, experience (≥6 months), and communication ability in using the local languages and familiarity with the local culture. Female providers were selected purposely because mothers can discuss their problems and concerns without fear or social stigma with female healthcare providers.

#### Mode of delivery

The intervention was administered to mothers in a face-to-face manner through group discussion and experience-sharing sessions. Although the healthcare facility does not offer PPD screening or intervention, the community has an awareness of the issues surrounding maternal mental health issues. Culturally, it is considered taboo to abandon postpartum mothers for 40 days after delivery to prevent PPD and other mental health problems. Thus, this approach helped the researchers to have sound group discussions and experience-sharing sessions. On occasion, the case scenario was used by the researchers as a trigger for group discussions and to increase participants' comprehension. Additionally, practical exercises were carried out. For instance, participants were asked to evaluate their current emotional feelings and look for potential reasons for those feelings, followed by discussions on how they handled the situation with the group members. Finally, a midwife discussed healthy ways of handling the situation.

#### Type(s) of location(s) where the intervention occurred, including any necessary infrastructure

Provider training was given in the Jimma University midwifery skill lab. Flip charts and LCD were used for the presentation. The intervention for pregnant mothers was delivered at the primary health facility in the same location where the participants were recruited and/or at the health center of the provider who delivered the intervention. Sometimes, the intervention was delivered in a health center room for inpatients, a meeting room, or any free room available by arranging the necessary infrastructure needed for the intervention.

#### Number of times the intervention was delivered and over what period, including the number of sessions, their schedule, and duration, intensity or dose

In total, 5 prenatal group sessions lasting 60 to 90 minutes were administered every week (for a total of 5 weeks).

#### Tailoring of the intervention

Mothers in both groups who had moderate-severe depression (PHQ-9 ≥ 10) were excluded from the study and were referred to a nearby hospital psychiatric outpatient department for further evaluation and treatment by a district clinician (psychiatrist). Similarly, mothers with moderate-severe depression (PHQ-9 ≥ 10) in both groups were referred to a nearby hospital psychiatric outpatient department at end-line data collection. Furthermore, mothers who were part of the intervention group were encouraged to visit a professional (a trained midwife or nurse) whenever they felt emotionally unstable and sought to assess their depressive status.

#### Modifications of the intervention during the study

No amendments were made to the intervention during the study.

#### How was adherence or fidelity assessed?

To assess providers' adherence to the intervention, pre- and post-test assessments were used followed by daily evaluations of the training. For pregnant women, a log frame was developed together with providers and supervisors to pinpoint the objectives and activities. Specifically, the objectives of the project, activities (number of sessions, content covered in each session), and attendance for each participant mother (number of mothers who participated in each session and finished each session correctly) were pointed out. Then, supervision was carried out at each health center following the log frame. Furthermore, interviews were held with some providers and participants about their satisfaction and feedback on the intervention.

#### Actual adherence or fidelity

Regarding the training of the providers, the average score on the pretest was 45%, but following the intervention (posttest), every provider had a score greater than 90%. About pregnant mothers' participation, all enrolled pregnant women finished all the sessions (100%), and all (100%) of the content intended to be covered in each session was covered. Moreover, at the end of the project, both participants and providers expressed their satisfaction and helpfulness with the intervention in their feedback.

### Outcomes

The major goal of this study was to determine the effectiveness of prenatal group-based psycho-education in preventing PPD and improving social support and postpartum literacy. In light of this, among the findings of this study, the first outcome was related to PDD prevention. Thus, the postpartum depression status of mothers was compared between the intervention and control groups, taking postpartum depression as the dependent variable. Mothers were assessed by the PHQ-9 tool during the 6-week postpartum period. It comprises 9 questions, each having scores of 0–3, with minimum and maximum scores of 0 and 27, respectively. Mothers who scored 0–9 were considered to have a normal score, and mothers who scored ≥10 were considered to have a depressed score. Additional factors that could influence the postpartum depression status of mothers were subsequently investigated. Similarly, using health centers as clustering variables, the independent variables were classified as level 1 (individual-level) or level 2 (community- or facility-level). A more in-depth list of variables, together with descriptions and measurements, is provided (See **Supplementary Table 2**).

### Sample size determination

The sample size was calculated based on the recommendations of sample size determination for cluster randomized controlled trials for a fixed number of clusters (46, 47) by using the following assumptions: the proportion of postpartum depression was 21.9% based on previous studies (10); 12% (effect size 0.12) reduction in the incidence of PPD; and the number of clusters available, 32 (16 clusters per arm), with a 95% confidence interval and 80% power, with an Intracluster correlation coefficient of 0.03558 (48). The sample size was calculated to determine the number of observations required per cluster for a two-sample comparison of proportions by using the normal approximation and G-power. Assuming individual randomization, the sample size per arm was 158. Then, to allow for cluster randomization, the design effect was calculated (DE = 1 + ρ (m − 1) = 1.7). Finally, the final sample size was determined by adjusting the fixed numbers of clusters, power, cluster size, and non-response rate.

Based on these findings, the average cluster size required for cluster randomization was 20, and the total sample size was 640 pregnant mothers, 320 in the intervention group and 320 in the control group.

### Data collection procedures and data quality control

The data were collected at 12–20 weeks of gestation and at 6 weeks after delivery. A well-validated, pretested, and structural questionnaire was used to obtain the data. The questionnaire was created after research on pertinent standard tools and the literature was conducted. The validity and reliability of each questionnaire were assessed as follows. Postpartum depression in mothers was assessed by the PHQ-9, which has shown good reliability and validity in screening for major depressive disorders among perinatal women in the study area (49–51). Similarly, social support was assessed by the Functional Social Support Questionnaire, and its validity and reliability were checked in the study area (52). Furthermore, for social support and self-esteem, a principal component was performed. Finally, the postpartum literacy and coping mechanisms of mothers were assessed by the Postpartum Depression Literacy Scale (PoDLiS) (53) and Brief COPE Scale (54), respectively. To that end, a factor analysis was performed on both variables. A more in-depth list of variables, together with descriptions and measurements, is provided. (See **Supplementary Table 2**).

For that purpose, an interviewer-administered structured questionnaire was adapted in English and then independently translated into two local languages, Oromo and Amharic, by language experts to ensure meaningful consistency. Training of the data collectors and eligibility screening were performed by a psychiatrist and principal investigator for 2 days. The data were collected by 8 midwives who were working in different health facilities other than the study area and were blind to the hypotheses of the study, the participants, and the cluster recruitment. The data collectors were supervised by two supervisors who had MSc in psychiatry and midwifery. A pretest was performed on 5% of the pregnant women who were in facilities outside of the study area before data collection. During the data collection, completed questionnaires were routinely reviewed for completeness, accuracy, and clarity on the spot. Before beginning each day's activities, any errors, ambiguity, incompleteness, or other difficulties encountered were identified, shared, discussed, or solved.

### Data analysis

Following the data collection, the data were cleaned, edited, coded, and entered into a computer with the help of Epidata Manager 4.4.1. The analysis was performed using the SPSS-25 statistical software packages. The data were analyzed using the intention-to-treat analysis approach. Postpartum depression was compared between the treatment and control groups based on the end-line results. Descriptive statistics were used to check for missing variables, errors, and outliers. Any errors identified at this step were corrected by revising the participant's response from the questionnaire. Moreover, descriptive analyses were carried out, and the frequency, mean, and standard deviation were used to describe the study population in terms of socioeconomic and demographic characteristics and psychosocial, obstetric, and other relevant variables.

The data were analyzed using a mixed-effects multilevel logistic regression model by considering both the cluster and individual levels. The multilevel approach allows for the evaluation of the effect of group- and individual-level variables on individual-level outcomes while accounting for observational independence within groups. Similarly, this model takes into account individual probability, which is statistically dependent on individuals' location. This context dependence was considered to generate correct regression estimations. Model fitness for the multilevel model was tested by using the log-likelihood ratio test. The influence of cluster-level variability on PPD was investigated using the intercept-only model and ICC. In addition, the variation between clusters was measured using the median odds ratio and proportional change in variance (55, 56).

Four models were built: the first was an empty model that was used to determine the extent to which cluster variation affects PPD. The second model was used for individual-level variables, the third for community-level variables, and the fourth for both individual- and community-level variables. Bivariate analysis was performed, and factors with a p-value < 0.25 were included in the second and third models. The final model included variables with a p-value < 0.05 in the second and third models. To determine statistical significance, a p-value < 0.05 was utilized; the AOR and 95% CI were used to indicate the strength of the association and the level of significance, respectively.

## Results

Of the 1,174 pregnant women screened for eligibility, 320 from the intervention site and 320 from the control site met the eligibility criteria. A total of 286 pregnant women enrolled in the intervention group, while 34 pregnant women declined to participate. A total of 264 pregnant women were enrolled in the control group, and 56 pregnant women declined. Five participants from the intervention site and seven from the control site refused to participate because they had busy work schedules. Similarly, 29 pregnant women from the intervention site and 49 pregnant women from the control site refused to participate because they had experienced discomfort related to pregnancy. None of the pregnant women's characteristics were significantly different between the two comparison groups. All of them were 12–20 weeks of gestational age and in the normal (score 0-4) and mild (score 5–9) ranges of depression status based on the PHQ-9 assessment results.

Finally, baseline data were collected, and an intervention was administered to 550 pregnant women (286 and 264 pregnant women from the intervention and control groups, respectively). However, nineteen (n = 19) pregnant women from the intervention group were lost to follow-up. Sixteen (n = 16) pregnant women went to their family of origin for delivery and postpartum care. All the participants participated in all the sessions of the intervention, and they were in good contact with the providers; however, we missed them during the end-line data collection time, whereas three (n = 3) pregnant women completely changed their home place; similarly, all the participants finished all the sessions. Similarly, twenty (n = 20) mothers in the control group were lost to follow-up. Seventeen (n = 17) pregnant mothers went to their family of origin for delivery and postpartum care, two (n = 2) moved their residence, and the remaining one (n = 1) died due to eclampsia at approximately 36 weeks of gestation.

**Figure 1:**
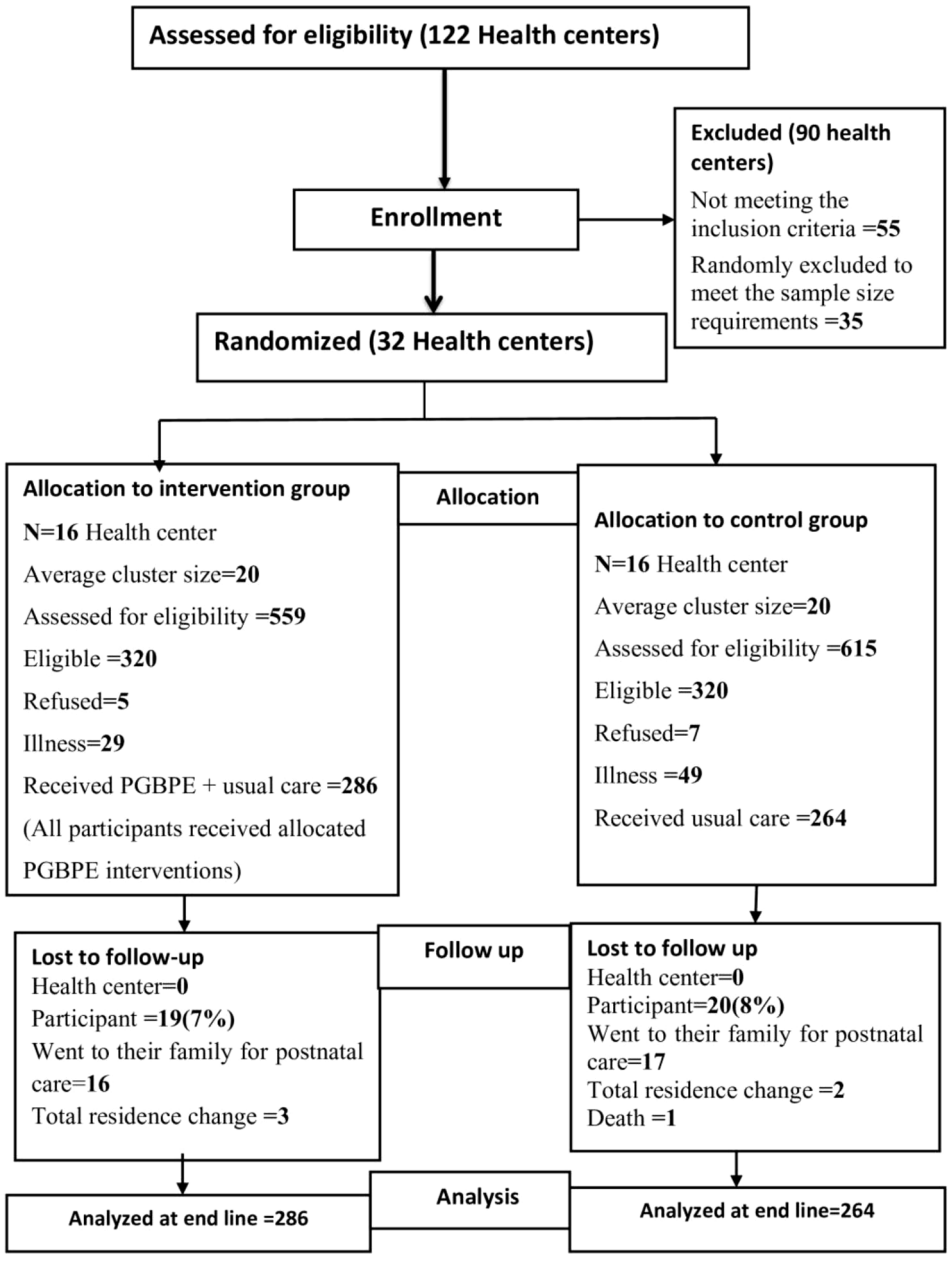
CONSORT 2010 trial flow chart, intervention study, Jimma Zone, 2023.

None of the participant characteristics related to loss to follow-up was significantly different between the intervention and control groups, and none of the reasons for loss to follow-up were related to the outcome of interest. Finally, endpoint data were collected from 511 participants: 267 (52.3%) from intervention clusters and 244 (47.7%) from control clusters. An intention-to-treat analysis was used for the final analysis. Finally, data analysis was performed for 550 mothers (from 32 clusters), taking into account 39 pregnant women who were lost to follow-up at the end. **Figure 1**. Trial flowchart of the study.

### Socio-demographic characteristics

Out of 550 pregnant women, more than half (52.9%) were 25–34 years old, with a mean age of 27.19 years (SD ± 5.7). Most of the pregnant women were in marital unions (484 [94.7%]; 521 [71.1%]). Two hundred and sixteen pregnant women (39.3%) did not attend formal education, whereas 204 (37.1%) had attained primary education (**Table 2**).

**Table 2:** Comparison of background variables, (N=550), (Interventions=286), Controls=264).

Variable with or without category	Intervention N (%)	Control N (%)	p-value
**Residence **			
Urban	164(57.3)	167(63.3)	.15
Rural	122(42.7)	97(36.7)	
**Age**			
15-24	86(30.1)	96(36.4)	.37
25-34	159(55.6)	132((50)	
=>35	41(14.3)	36(13.6)	
**Marital status**			
Married	272(95.1)	249(94.3)	.54
In-relationship	10(3.5)	9(3.4)	
Others*	4(1.5)	6(2.4)	
**Education status**			
informal education	125(43.7)	91(34.5)	.04
Primary (1-8)	104(36.4)	100(37.9)	
Secondary (9-12)	35(12.2)	56(21.2)	
College/above	22(7.7)	17(6.4)	
**Job**			
Housewives	206(72)	185(70.1)	.67
Private works	42(14.7)	44(16.7)	
Gov’t employs	27(9.4)	22(8.3)	
Domestic workers	11(3.8)	13(4.9)	
**Household income**			
<3000	147(51.4)	122(46.2)	.26
3001-5000	76(26.6)	77(29.2)	
>5000	63(22)	65(24.6)	
**Parity**			
Primipara	198(69.2)	146(53.3)	.00
Multipara	88(30.8)	118(44.7)	
**Unplanned pregnancy for the mother**			
Yes	65(22.7)	58(22)	.81
No	221(77.3)	206(78)	
**Unplanned pregnancy for the father**			
Yes	71(24.8)	59(22.3)	.49
No	215(75.2)	205(77.7)	
**History of mental health**			
Yes	14(4.9)	10(3.8)	.56
No	272(95.1)	254(96.2)	
Social support			
Adequete	63(22.03)	60(22.73)	.84
Inadequete	223(77.97)	204(77.27)	
PPD Literacy			
Literate	78(27.27)	77(29.17)	.62
Illiterate	208(72.73)	187(70.83)	

Others*=divorced, separated, widowed.

### Effectiveness of group-based psycho-education in preventing postpartum depression

The findings of the study revealed that group-based psycho-education offered by primary healthcare providers at PHCUs was effective at preventing PPD. Postpartum depression was significantly lower in the intervention clusters than in the controls (20 (7.6%) *vs*. 74 (28.9%), p < 0.001)/65% (AOR = 0.35, 95% CI = 0.13–0.99). Similarly, the phi (φ) coefficient = −0.279 with df=1 indicates a very strong effect size (57), while no difference was observed at baseline. Furthermore, for the primary outcome variable, the relative risk and absolute risk factors were calculated. Mothers receiving prenatal group-based psycho-education had 25% of the PPD risk of mothers receiving the usual care [relative risk difference: 0.25(0.07/0.28)]; similarly, prenatal group-based psycho-education reduces the absolute risk of PPD by 21% as compared to usual care [absolute risk difference: 0.21(0.28–0.07)].

### Factors associated with postpartum depression

To assess the model's applicability, an empty model of mixed-effects multilevel logistic regression was initially conducted, which revealed that, of the total variation in mothers' PPD status across clusters (health centers), 19.4% (Intracluster correlation coefficient (ICC) 19.4%, p-value < 0.01) was attributed to the cluster level. Similarly, a median odds ratio (MOR) of 2.3 (which differs from 1 and shows no association) indicated the occurrence of area-level variation in mothers' PPD status, indicating that the dataset was a good fit for mixed-effects multilevel logistic regression. A more detailed assessment of each of the four models is presented in **Table 3**.

**Table 3:** Random intercept model/measure of variation.

Measure of variation	Model 1	p-value	Model 2	p-value	Model 3	p-value	Model 4	p-value
Variance (SE)	0.79 (0.32)	<0.01	1.26 (0.53)	<0.01	0.75 (0.44)	<0.04	0.99 (0.51)	<0.02
ICC (%)	19.4%		27.7%		18.6%		23.1%	
MOR	2.3		2.9		2.3		2.6	
PCV	Reference		59.5%		5.1%		25.3%	
Deviance	−978							
LR test *vs*. logistic model (p-value)	<0.02							

SE, standard error; ICC, Intracluster correlation coefficient; MOR, median odds ratio; PCV, explained variation; Model 1: intercept only, Model 2: adjusted for individual-level variables, Model 3: adjusted for community-level variables, Model 4: adjusted for individual- and community-level variables.

Finally, five variables at the individual level (second model) and five at the community level (third model) with p-values ≤ 0.05 were included in the fourth model (final model). According to the final model (fourth model), group-based psycho-education intervention, social support, an emotional relationship with an intimate partner, PPD literacy, self-esteem, job, dysfunctional coping mechanisms, and neonatal complications were significantly associated with PPD.

Mothers who were under the intervention clusters and received PGBPE (AOR = 0.35, 95% CI = 0.13–0.99) were less likely to develop PPD as compared to those who were under the control arm and received the usual care. Mothers who had good social support (AOR = 0.04, 95% CI = 0.01–0.15) were less likely to develop PPD. Mothers who had partner emotional support (AOR = 0.024, 95% CI = 0.12–0.51) were less likely to develop PPD. Mothers who had good PPD literacy (AOR = 0.25, 95% CI = 0.11–0.62) were less likely to develop PPD. Mothers who had high self-esteem (AOR = 0.22, 95% CI = 0.11–0.47) were less likely to develop PPD. On the contrary, mothers who were domestic workers (AOR = 9.75, 95% CI = 3.37–28.16) were more likely to develop PPD as compared to those who were housewives. Mothers who had neonates with complications (AOR = 5.79, 95% CI = 2.04–16.45) were more likely to develop PPD. Mothers who used unhealthy coping mechanisms (AOR = 2.39, 95% CI = 1.06–5.42) were less likely to develop PPD (**Table 4**).

**Table 4:** Multilevel logistic regression analysis (N=550).

Variable	Model 2 AOR (95% CI)	Model 3 AOR (95% CI)	Model 4 AOR (95% CI)
**Individual-level factors**			
**Age**			
15–24	**1**		
25–34	0.64(0.19–2.17)		
≥35	0.69(0.33–1.47)		
**Job**			
Housewife	1		1
Private employee	3.79(1.1412.69)***		3.13(1.11–8.85)*
Gov’t employee	0.37(0.12–1.23)		0.39(0.08–1.84)
Domestic worker	8.97(2.66–30.26)*		9.75(3.37-28.16)***
**Parity**			
Primipara	1		
Multipara	1.382(0.68–2.82)		
**Unwanted pregnancy (mother)**			
Wanted	1		
Unwanted	1.19(0.62–2.27)		
**Birth outcome**			
Normal	0.42(0.13–1.34)		
Not normal	1		
**Exclusive breastfeeding**			
Yes	0.67(0.34–1.43)		
No	1		
**Stressful life in the last 12 months**			
Yes	1.08(0.53–2.21)		
No	1		
**History of mental health**			
Yes	2.49(0.64–9.75)		
No	1		
**Loneliness**			
Yes	3.08(1.47–6.46)*		1.89(0.68–4.74)
No	**1**		1
**Self-esteem**			
High	0.31(0.19–0.53)***		0.22(0.11–0.47)***
Low	**1**		**1**
**Emotional coping**			
Yes	0.68(0.35–1.34)		
No	1		
**Problem-based coping**			
Yes	0.71(0.28–1.82)		
No	1		
**Dysfunctional coping**			
Yes	3.67(2.04–6.57)***		2.39(1.06–5.42)*
No	**1**		**1**
**Postpartum depression literacy**			
Good	0.16(0.06–0.42)***		0.25(0.11–0.62)*
Poor	**1**		**1**
**Community-level variables**			
**Residence**			
Urban		1	
Rural		2.03(.96–4.30)	
**Marital status**			
Married		1	
In-relationship		1.00(0.07–14.50)	
Others (divorced, separated, widowed)		2.46(0.68–8.94)	
**Group-based psycho-education**			
Yes		0.28(0.11–0.69)***	0.35(0.13–0.99)*
No		**1**	**1**
**Social support**			
Adequate		0.03(0.01-0.13)***	0.04(0.01–0.15)***
Not adequate		**1**	**1**
**Emotional relationship with partner**			
Good		0.36(0.19–0.68)*	0.24(0.12–0.51)***
Poor		**1**	**1**
**Childhood abuse**			
Yes		1.46(0.66–3.21)	
No		1	
**Support from her mother**			
Good		0.99(0.36–2.75)	
Poor		1	
**Unwanted pregnancy (partner)**			
Wanted		1	
Unwanted		1.74(0.75–4.07)	
**Intimate partner violence**			
Yes		2.46(1.28–4.71)*	1.60(0.71–3.66)
No		1	1
**Neonatal complications**			
Yes		5.13(1.85-14.20)*	5.79(2.04–16.45)*
No		**1**	**1**
**Child sleeping problem (crying)**			
Yes		2.46(0.63–9.33)	
No		1	

*p<0.05, ***p<.0001.

## Discussion

The findings of this study revealed that prenatal group-based psycho-educational interventions offered by maternal healthcare providers in PHCUs were effective at preventing postpartum depression. Mothers who were assigned to intervention clusters and who received prenatal group-based psycho-education were protected from PPD compared to mothers in the control clusters who received routine care. Our study follows a PPD prevention strategy, and only pregnant women with non-depressed and mild depressive symptoms and 12–20 weeks of gestation were included in the study because at this time, both the psychosocial and biological vulnerability of the mother to stress are very low (58, 59). Stable emotional status helps participants to effectively follow interventions, understand the effect of PPD, and prepare themselves for healthy coping. The study is consistent with a systematic review that showed a preventive effect for PPD by evaluating primary prevention strategies which included only the non-depressed women during pregnancy, and their families (22, 38).

Moreover, prevention strategies, which are offered for non-depressed pregnant women and are the most cost-effective, less stigmatizing, and more likely to be used by participants, were effective (22). Maternal healthcare providers can easily apply them to community or primary healthcare facilities. Similarly, in this study, a maternal healthcare provider was used for the implementation of the study. This is consistent with a systematic review of a study that shows the effectiveness of community and facility-based intervention by maternal health care workers (22, 26). As such, this trial is original and makes an important contribution to research on PPD and should be applied in community-based settings to devise policies regarding strategies to prevent postpartum depression.

Furthermore, our study shows that mothers who had adequate social support and had good emotional relationships with their partners were protected from PPD. Based on the stress process and stress vulnerability model (2, 21), and evidence from the study area, we used inadequate social support (28, 38, 42) as stressors, and adequate social support as a mediator and included partner or family as part of the intervention. So, one of our psycho-education targets was improving the social support of pregnant women. In line with these findings, empirical evidence affirms the mediating role of social support, specifically partner support, between psychiatric vulnerability and life stress (26, 30). Similarly, a follow-up study showed that partner support protected mothers from PPD by dampening the rise in corticotropin-releasing hormone during pregnancy (31). Furthermore, the intervention involved family and was given in groups, and the majority of common questions raised by pregnant women were addressed predominantly through discussion and experience sharing within the groups, which helped the pregnant women feel like they were not alone and more confident in the group's support. The results were broadly consistent with studies (30, 34) which suggested that interventions to prevent postpartum depression are more likely to be successful if they are initiated during pregnancy, involve family support, and are group-based.

Similarly, in this study, mothers who had high self-esteem were more likely to be protected from PPD. This implies that having positive social relationships helps mothers buffer the effects of psychiatric vulnerability and life stress. Furthermore, in this study, mothers who were domestic workers were more likely to develop PPD than those who were housewives. In line with these findings, evidence shows that this commonly occurs because mothers have multiple burdens, such as economic problems and workload, and because the pregnancy itself may not be wanted or supported (60). Thus, PPD is an indication of distress in mothers who perceive social isolation from their partner, family, or community (28).

In this study, mothers who had good PPD literacy were protected from PPD compared to mothers with low PPD literacy. Relatedly, evidence indicates that having accurate knowledge about mental health problems is the first step toward help-seeking behavior (61). Indeed, self-help strategies such as preparing positive social support, good sleep, and nutrition are aspects of PPD literacy that help mothers cope with stress and prevent the risk of PPD (62). Generally, PPD literacy helps mothers take early action and protect themselves from PPD through health coping methods.

The findings of the present study also showed that mothers who used dysfunctional coping strategies during stressful situations were exposed to PPD. This implies that mothers' coping skills help them protect themselves from the development of mental health problems. As noted, mothers' coping skills involve simple efforts to resolve personal and interpersonal issues, which are the main causes of depression, and seek to reduce or tolerate stress or conflict (63). However, sometimes, individuals use this skill more inappropriately with less adaptive coping strategies such as self-blame and rejection of truth, which are highly associated with PPD (63, 64). The findings of the study also revealed that mothers whose neonates had complications developed depressive symptoms compared with mothers whose neonates had no complications. Other related findings have revealed that mothers of newborns admitted to the neonatal intensive-care unit (NICU) have persistently greater incidences of PPD than mothers of healthy-term infants outside the NICU (65). Pertinently, the findings of the present study indicated that postpartum depression is linked to mothers' psychosocial problems. Generally, these findings pinpointed that providing social support for childbearing women is an important intervention for preventing postpartum depression.

### Strengths/implications/limitations

To the best of our knowledge, this study can be considered new in the Ethiopian context, and this would make it the first preventive interventional study. Thus, the findings can offer fresh insights into the effectiveness of group-based psycho-education interventions for preventing postpartum depression by first-line healthcare providers in primary healthcare facilities. Since 2012/13, the Ethiopian National Mental Health Strategy has mandated that mental health be incorporated into the primary healthcare system. It has been guaranteed that persons in need of services should have access to care as close to home as possible and in the least restrictive setting. The plan is also designed to help health workers learn competencies at different levels of care so that they can easily recognize, monitor, and manage mental health conditions. As a result, as an added value, the existing health system should scale up this strategy. Thus, this study identified effective ways of assessing and preventing PPD at the primary maternal healthcare level and creating links between mothers and mental health services. Therefore, the Federal Ministry of Health and Jimma Zone Health Office should consider this strategy when promoting maternal mental health in the primary healthcare setting.

Moreover, these findings will not only add new knowledge to the existing knowledge but also help policymakers and other concerned stakeholders to prepare relevant resources, flourish their maternal healthcare programs, and prevent PPD. Equally important, the results of the study can be used by other investigators who want to conduct further research.

This study has certain limitations. First, since the data collection was performed through face-to-face interviews, participants may have provided incorrect information about their personal and family lives. As a result, the data's reliability was limited and relied on the information gathered by the interviewers. Second, because the PHQ-9 is based on self-reports, the data generated with this tool might differ from what was truly observed by specialists. Furthermore, mothers with moderate to severe depression (PHQ-9 ≥ 10) were excluded from the trial, and the impact of the intervention was not assessed throughout pregnancy.

The cluster randomized control trial may also have limitations in terms of internal and external validity. Participants' selection bias may pose a threat to internal validity. However, we used some strategies to minimize such bias and improve internal validity. We created strict inclusion criteria for both clusters and participants. Then, based on the criteria, we selected 32 non-adjusted health centers that had adequate buffer zones between them to reduce information contamination. Finally, before randomizing clusters into intervention and control groups, we recruited participants.

Furthermore, our study was preventive; we selected non-depressed pregnant women to prevent PPD before its onset. For this purpose, we further selected pregnant women 12–20 weeks of gestational age. Because, at this stage, depression is less likely to occur due to low biological and psychosocial burdens, however, we can still generalize this finding to pregnant women 12–20 weeks of gestation with normal and mild depressive symptoms who attend ANC in primary health care facilities. Furthermore, since the outcome is sensitive to change (proportion), the loss of follow-up in this study was 49/550 × 100 = 8.9%. We conducted an intention-treat analysis, and a loss to follow-up of less than 20% cannot cause a significant validity problem (66). The trial was single-blind; therefore, only the outcome assessors were blinded, whereas participants and providers were actively involved in the intervention process. In addition, discomfort related to pregnancy and the mother's places of delivery were not exclusion criteria. Thus, this situation has caused a decrease in participation and an increase a loss of follow-up.

## Conclusions and recommendations

The findings of this study revealed that group-based psycho-education offered by primary healthcare providers at primary healthcare facilities was effective at preventing PPD. The integration of this intervention into the healthcare scheme in maternal healthcare service can prevent PPD and help to promote maternal mental health. Thus, it is suggested that midwives and other health personnel working in the primary care system be introduced to and educated on this intervention strategy. In addition, to enhance the contribution of the intervention, additional work should be done particularly on the long-term effect of the intervention, and to enhance partner support and mothers' healthy coping skills during stressful situations. Therefore, in line with the moves being taken by the Ethiopian Ministry of Health to promote mental health in primary healthcare facilities, this trial should be taken into account for broader implementation.

## Data Availability

The original contributions presented in the study are included in the article/**Supplementary Material**. Further inquiries can be directed to the corresponding author.
