# Supplementary material for: Effectiveness of group-based psycho-education on preventing postpartum depression among pregnant women by primary healthcare provider in primary healthcare institution: a cluster-randomized controlled trial
Source: Front Psychiatry. 2024 Sep 10;15:1433942. doi: 10.3389/fpsyt.2024.1433942 (PMC11420118; doi:10.3389/fpsyt.2024.1433942)
Supplement: Supplementary file 1 [file DataSheet1.zip › Prenatal Group-Based Psychoeducation Manual for Providers.pdf]

PRENATAL GROUP-BASED PSYCHOEDUCATION FOR PROVIDERS

Hero mom

Jimma University Institute of Health

Faculty of Health Science School of Public Health

Department of Population and Family Health

September, 2021

Jimma University

Acknowledgement

This prenatal group-based psych education Manual has been prepared for primary health care provider in preventing postnatal depressions. We would like to acknowledge the contributors of Prof. Zewdie Birhanu(Health, Behavior and Society), Dr. Muluemebet Abera (Associate professor of reproductive health), Dr.Mathios Mesaane (Psychiatrist),Yonas Tesfaye(Assistant Prof., in Psychiatry Nurse), and Bonsa(Assistant Prof., psychologist), Biru abdisa(Assistant Prof. and Marta Tessema(MSc, in clinical Midwifery,PhD.Fellow) from Jimma University.

Table of content

Contents

Acknowledgement I
Table of content I
Welcome, expectations, objectives I
Materials and preparation I
Session 1: Perinatal Depression and Baby Blue 1
Session Two:-Postpartum Depression screening and Psychosocial risk assessment 6
Session Three; Group Based Psycho-Education 12
Session Four; - Prevention Methods 14
Session Five: Social Support and Partner Invitation 29
Annexes 36
  Training evaluation form 36
  Pre-test/Post-test evaluation 37
  Reference 38

Welcome, expectations, objectives

Materials and preparation

Flip chart, note cards, markers, pen. Flip chart with learning objectives (covered until presented at the end of the session)

Time: 20 minutes

Activity

1. Open the session and welcome participants. Review the training schedule.

2. Ask each participant to introduce themselves and agree on one expectation they share for the training. Facilitators should write each expectation on a flip chart sheet.

3. Compare participant expectations to training objectives.

At the end of the training the participant will be able to:-

Describe postpartum depression, its risk factors, symptoms, and consequences for the mother child and family as a whole.

Differentiate postpartum depression and baby blue

Screen postpartum depression using patient health quationar-9

Facilitate group-based psycho-education

Describe healthy coping and prevent postpartum depression

Describe social support, its benefit for prevention of postpartum depression

Help pregnant women social support for this perinatal period

Help pregnant women to prevent postpartum depression

Session 1: Perinatal Depression and Baby Blue

Objectives

- Describe perinatal depression/mental health during postpartum period
- Discuss how its seen in our community ,beliefs, stigma behind PPD

Materials and preparation

- Flip chart
- Markers

Time 60-90 minute

Activity /Discussion:

1. Ask: What is perinatal mental illness/depression?
2. Listen to their responses and then share the following:

Perinatal mental illness refers to psychiatric disorders that are prevalent during pregnancy and as long as 1 year after delivery. There are hundreds of mental illnesses but the Commonest type found in the perinatal period are: -

- **Perinatal depression: -** it's up to 63%
- **Perinatal anxiety: -** It's up to 20% It can happen together with depression
- **Perinatal psychosis: -**rare case 1or 2/1000 births but if, occurs very dangerous for the women, she may kill her baby.
- **Postpartum blues**: -it occurs the 1st 10 days after delivery and prevalent up to 84% of women

Because depression is the commonest mental health disorder during perinatal period research on untreated mental health disorder is often based on studies of depressed women. PPD is a clinical condition that lasts for at least two weeks, creates significant impairment in functioning, and typically requires professional treatment (DSM-IV-TR); Am. Psychiatric. Assoc. 2000).

DSM-5 defines PPD as sub-category of major depressive disorder. It doesn't define PND as a discrete disorder. (DSM-V,2013). It is a term used for major depressive episodes during pregnancy and/or after the birth or adoption of a baby.

3. Ask: What are the cause's perinatal depressions?

4. Listen to their responses and then share the following:

In many cultures, both medical and traditional explanations are used to understand the causes of ill health. Traditional models are often related to spiritual or supernatural causes, such as bad spirits or witchcraft. It is important to be aware of the beliefs about the causes of mental illness in a specific culture. However, one should also be aware of the medical theories and use these theories to explain mental illness to the people while working in a medical clinic. It is useful to keep in mind the following main factors that can lead to mental illness:

Biological

- In the life span of a healthy female mood fluctuation related with neuroendocrine events (e.g., menstruation, puberty, menopause and peripartum period). Reproductive hormones increase over the course of pregnancy to a degree that is unparalleled by any other neuroendocrine events. Mood disturbance is associated with the sudden withdrawal of estrogen, estrogen fluctuations, and sustained estrogen deficie (Douma et al. 2005). The mood fluctuation present within one to two weeks after delivery is called post-partum blue. Its prevalent up to 84% of women and it can resolve by itself within this period of time without intervention. (Michael W et al.2014, WHO 1992). But Postpartum depressions is generally independent of the blues, but the postpartum blues do represent a risk factor for postpartum depression (O'Hara WM.et al.1991). **Stress hormones: -** follow a pattern similar to reproductive hormones, such that they increase over the course of pregnancy and then drop after delivery. Such as corticotropin releasing hormones (CRH), adrenocorticotropic hormone (ACTH), β-Endorphin, Cortisol, Catecholamines hormones etc. higher levels of Stress hormones in mid-to-late pregnancy is predictive of PPD symptoms during the first few postpartum months. Again, during Pregnancy and in the first few post-partum days, the innate immune system (proinflammatory cytokines and anti-inflammatory cytokines) will increase and associated with PPD symptoms. Finally, *Heredity or genes.* Heredity is an important factor for several mental disorders. However, if one parent has a mental illness, the risk that the

children will suffer from a mental illness is very small. This is because, like diabetes and heart disease, these disorders are also influenced by environmental factors.

Psychosocial

In contrast, psychological models such as the stress process model (Pearlin et al. 1981) and the cognitive behavioral model of PPD (O'Hara et al. 1982) emphasize the deleterious role of psychological stressors (e.g., father abandonment, financial strain) and underlying cognitive vulnerabilities (e.g., negative attributional style) and the ameliorating role of psychosocial resources (e.g., social support, self-esteem). These theories posit that pregnancy, childbirth, and new parenthood are stressors for many mothers, helping to explain why women may be especially vulnerable to depression at this life stage.

Biological and psychological theories have guided research and provided insight into an important piece of the PPD puzzle, but they do not help us understand how psychosocial stress processes are instantiated in women's brains and bodies, nor how genetic or epigenetic changes interact with psychosocial risk factors to influence PPD risk. To bridge this divide, integrated models have been developed, including the stress vulnerability model, which proposes that stress can trigger PPD in women with genetic, hormonal, and cognitive vulnerabilities (O'Hara et al. 1991). There is evidence to support this theory (e.g., Ross et al. 2004), although most studies have relied on family and personal history of depression as proxies for underlying genetic risk. More recently, Halbreich's (2005) bio-psycho-social-cultural model overlaid the stress vulnerability model with additional biological sophistication and added cultural aspects to the list of moderating variables. By this account, biological vulnerability is conceptualized as a genetically derived hypersensitivity to hormonal changes and to dysregulation or impaired adaptation mechanisms in the central nervous system. This vulnerability is thought to interact reciprocally with the environment, both shaping the organism's responses to environmental challenges and being shaped by stressors and positive experiences over the life span.

General Risk Factors For Postpartum Depression	
**During pregnancy**	**During post-partum period**
• Adolescent pregnancy<br>• Unwanted pregnancy<br>• Being unmarried or separated<br>• Marital relationship: unsupportive; polygamous<br>• Lack of practical support<br>• Spouse/domestic violence<br>• Previous stillbirth or repeated miscarriage<br>• Nulliparity<br>• Poverty and lack of financial resources<br>• Pregnancy as a result of rape<br>• Difficult relationship with in-laws<br>• Antenatal depression or severe anxiety<br>• Illnesses in this pregnancy, antenatal hospital admission, Past psychiatric history	• Adolescent pregnancy<br>• Unintended pregnancy<br>• Unmarried<br>• Difficulties with husband's behavior (physical violence; verbal abuse; alcohol use; being illiterate and unemployed; providing little assistance; rejecting the pregnancy)<br>• Inability to confide in partner<br>• Poverty (low income; lack of personal income generating activity; inadequate housing)<br>• Overcrowding and lack of privacy<br>• Antenatal depression or severe anxiety<br>• Illnesses during pregnancy, antenatal hospital admission, operative birth<br>• Past psychiatric history<br>• Large number of children<br>• Infant unsettled, sick, not thriving<br>• Problematic relationship with in-law family (mother-in-law and sister-in-law)<br>• Birth of a girl child in cultures over-valuing boy child<br>• Lack of sustained, dedicated, practical care after birth for the culturally prescribed period and Other stressful life events

5. Ask: What is the impact of PPD

6. Listen to their responses and then share the following:

Impact of Perinatal Depression

Perinatal depression is particularly important b/c it occurs at a critical time in the life of women, her baby and her family. Failed to treat promptly may result prolonged negative effect on the mother, the r/p between mother and baby and the child psychological, social and educational developmental. The relationship between mother and her partner also negatively affected

Mothers are gift for her husband, children, family and community as a whole, she has a potential to do everything, she is multi tasker, she can protect herself and her family from any problem even more than male can. But she should be healthy and have the capacities and skills to protect herself both from physical and emotional problems. Even children learn more strength and life skills from their mothers than their fathers. ''Teaching mother is teaching family or community''. Evidence indicates that the emotional problem of the women: depression and significant depressive symptoms, carries adverse physical and psychological consequences for both the mother, child, and families.

IMPACT ON THE MOTHER

FUNCTIONAL IMPAIRMENT: - Depression affects women decision-making abilities and causes feeling of overly anxious, doubting her ability to care of her family. Not responding babies need appropriately and See baby's behavior is difficult, difficulty of exclusive breast feeding. Fail to recognizing babies' cues that have potential impact on the development of the baby

RELATIONSHIP IMPAIRMENT: - irritability, withdrawing from loved ones, feeling distant from her baby and thinking about hurting herself or her baby. This abnormal behavior and irritability of the women affects her interpersonal relationship and results couple dysfunctions, intimate partner violence and child abuse.

CLARITY OF THINKING: -Maladaptive behaviors and disability in the areas of clarity of thinking, low self-esteem, poor cooping ability to any stress and lack of interest for everything. Negative view of motherhood

SELF-CARE: -Depression also affects a woman healthy life styles causes loss of appetite and poor nutrition, lack of sleep, lack of adequate rest. Low health responsibility for herself and baby. not following pregnancy care, Its linked to higher rates of spontaneous abortion, prolonged labour and operative deliveries. Risk of Suicide, substance abuse, risk of depression for next pregnancy

Summary

In Study Session 1, you have learned that:

1. Health is a broad concept containing several different aspects. Physical and mental health issues are often interrelated and wellness is expressed through the integration of mental, physical, emotional, spiritual and social health components.
2. Maternal mental well-being is really important for you as maternal health practitioner because it affects a women health, functionality, clarity of thinking and self-care. And it has great impact on mothers, children, partner, family, community and health care system.

Session Two:-Postpartum Depression screening and Psychosocial risk assessment

Objective:-

At the end of these sessions the trainee should be able to:

1. Explain diagnosis method of perinatal depression
2. Explain its difference with baby blue
3. Discuss possible risk assessment technique
4. Apply PHQ-9 and ANRQ for perinatal depression
5. Apply evidence-based intervention based on PHQ-9 and ANRQ result

Materials and preparation

- Flip chart
- Markers, DSM-5, PHQ-9 Tool, PNRQ (postnatal Risk Assessment tool**)**:

Time 4 hrs.

Diagnosis method of perinatal depression

The gold standard for diagnosing PPD is a clinical interview, the most well-known of which is the Structured Clinical Interview for the DSM-IV (First et al. 2002). There are also shorter screening tools commonly used in research.

Based on DSM-5

- Five (or more) of the following symptoms have been present during the same 2-week period and represent a change from previous functioning:

A. At least one of the symptoms is present either:

(1) depressed mood or

(2) loss of interest or pleasure.

B. The symptoms cause clinically significant distress or impairment in social, occupational, or other important areas of functioning.

C. The episode is not attributable to the physiological effects of a substance or to another medical condition.

DSM-5	
1. Depressed mood most of the day, nearly every day,	6. Recurrent thoughts of death (not just fear of dying), recurrent suicidal ideation without a specific plan, or a suicide attempt or a specific plan for committing suicide.
2. Markedly diminished interest or pleasure in all, or almost all, activities most of the day, nearly every day	7. Fatigue or loss of energy nearly every day.
3. Significant weight loss when not dieting or weight gain (e.g., a change of more than 5% of body weight in a month), or decrease or increase in appetite nearly every day.	8. Feelings of worthlessness or excessive or inappropriate guilt nearly every day
4. Insomnia or hypersomnia nearly every day.	9. Diminished ability to think or concentrate, or indecisiveness, nearly every day

5. Psychomotor agitation or retardation nearly every day	

Screening

Estimated Time: 10-15 minutes

Format: Individual

Screen Outline

a. Introduce self and screening program

b. Review Screen Results

Screen with PHQ-9

a. Introduce self and screening program

Hello, my name is ___________ and I am xxx [relationship to practice].

As a practice, our job is to help mothers get a good start for their babies. To help us support you, we'd like you to take some time to complete a form about your mood over the last couple of weeks. If you prefer to go through them together, we can do that.

**Assessment of depression: - Patient health questioner -9(PHQ-9)**					
**Over the past 2 weeks, how often have you been bothered by any of the following problems?**					
**S/R**	QUESTIONS	Not at all	Having several days	More than half of the days	Nearly every day
**1**	Little interest or pleasure in doing things	0	1	2	3
**2**	Feeling down, depressed or hopeless	0	1	2	3
**3**	Trouble falling asleep, staying asleep, or sleeping too much	0	1	2	3
**4**	Feeling tired or having little energy	0	1	2	3
**5**	Poor appetite or over eating	0	1	2	3
**6**	Feeling bad about yourself, or that you're a failure or have let yourself or your family down	0	1	2	3
**7**	Trouble concentrating on things, such as reading the newspaper or watching television	0	1	2	3
**8**	Moving or speaking so slowly that other people could have noticed. Or, the opposite — being so fidgety or restless that you have been moving around a lot more than usual.	0	1	2	3
**9**	Thoughts that you would be better off dead or hurting	0	1	2	3

	yourself in some way				
	Column total				
	Add totals together				
**10**	If you checked of any problems, how difficult have those problems made it for you to do your work, take care of things at home, or get along with other people?				
	_____ Not difficult at all _____ Somewhat difficult _____ Very difficult _____ Extremely difficult				

Over the last 2 weeks, how often have you been bothered by any of the following problems?

Not at all	Several days	More than half the days	Nearly every day

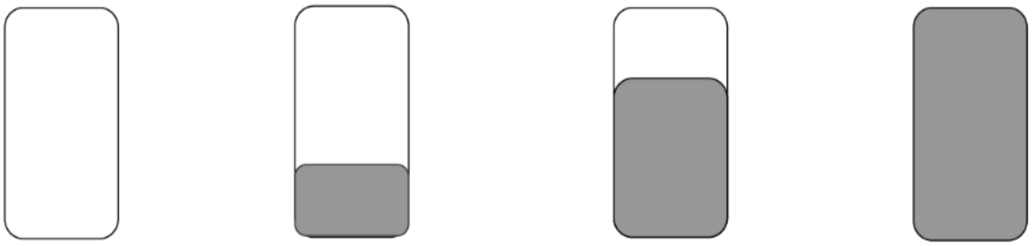

B. Review Screen Results

Let's review the results.

Low Risk: PHQ-9 < 10 Looks like things are generally going okay for you. We will check in on your mood again in 6 months. However, should you experience any changes that are of concern, please do not hesitate to bring it up with me or anyone in the practice.

Crisis Management: If mother scores > 19 on the PHQ-9 or endorses item 9 on PHQ, or presents with high risk indicators (e.g., psychotic symptoms, thoughts of harming baby), follow clinic protocol and involve a behavioral health specialist (in our case refer to psychiatric clinic)

For women whose PHQ-9 is between 10-19: Your score suggests that things might be difficult at this time. This is common among mothers of new babies. Let's schedule a follow-up appointment with professional

Introducing the PNRQ (Risk Assessment):

Thank you for meeting with me. I reviewed the form you filled out about your mood at your last appointment. I appreciate how honest you were with your responses. [REPEAT PHQ-9 if it has been more than 2 weeks since it was last completed]. Before we decide on a care plan, I'd like you to complete a different set of questions about your personal history so we can decide how best to help you. We can fill it out together if you prefer.

	Part III: psychosocial risk assessment<br>**Instructions:** Please circle numbers 1-6 or tick yes/ no, as applicable and complete all items.						
301	Have you ever had a period of 2 weeks or more when you felt particularly worried, miserable or depressed?	*Yes* ☐		*No* ☐			
301a	If yes, did this: Seriously interfere with your work and your relationships with friends and family	*Very much*		*Some what*		*Not at all*	
		1	*2*	*3*	*4*	*5*	*6*
301b	Lead you to seek professional help? If yes, did you see a psychiatrist () psychologist/counselor () GP/doctor ()	Name of health professional: ---------					
301c	Did you take tablets/herbal medicine?	*If yes, please specify:* ------------------					
301d	Do you have any other history of mental health problems? (i.e. Eating disorder, Psychosis, Bipolar disorder, Schizophrenia)<br>*If yes, please specify:* -----------------------------	*Yes* ☐		*No* ☐			
302	Is your relationship with your partner an emotionally supportive one? If you have no partner circle 6)	*Very much*		*Some what*		*Not at all*	
		1	2	3	4	5	6
303	Have you had any stresses, major changes or losses in the last 12 months (e.g. separation, domestic violence, unemployment bereavement or migration)?	*Yes* ☐			*No* ☐		
	If yes, please specify: -------------------						
303a	How distressed were you by these stresses, changes or losses?	*Very much*		*Some what*		*Not at all*	
		1	*2*	*3*	*4*	*5*	*6*
**304**	Would you generally consider yourself a worrier?	*Very*		*Some*			*Not*

		much		*what*			*at all*
		1	*2*	*3*	*4*	*5*	*6*
**305**	In general, do you become upset if you do not have order in your life (e.g. regular time tablet, a tidy house)?	*Very much*		*Some what*		*Not at all*	
		1	*2*	*3*	*4*	*5*	*6*
**306**	Do you feel you have people you can depend on for support with your baby?	*Very much*		*Some what*		*Not at all*	
		1	*2*	*3*	*4*	*5*	*6*
307	Were you emotionally abused when you were growing up?	*Yes*	☐		*No*	☐	
**308**	Have you **ever** been abused sexually ( ) or physically ( )?	*Yes*	☐		*No*	☐	
**309**	When you were growing up, did you feel your mother was emotionally supportive of you?	*Very much*		*Some what*		*Not at all*	
	Do you have any other concerns that you would like to talk about today?-- --						

AT NEXT APPOINTMENT:
Give the ANRQ or review results of PHQ-9 and PNRQ risk composite with woman.

Moderate Risk Profile: PHQ-9 ≥ 10 and ANRQ < 24: Your responses suggest that things might be stressful for you right now. At the same time, you have a number of strengths. Proceed with Engagement/Education/Linkages.

High Risk Profile: PHQ-9 ≥ 10 and ANRQ ≥ 24 Your responses suggest that this might be a stressful time for you. In addition, there appears to be a number of personal factors [highlight them] that put you at risk for postpartum depression.

Summary Session Two

1. All mothers should be assessed for PND and ANRQ at first ANC visit by PHQ-9 and by ANRQ.
2. The result should be given and discussed with the mother

Session Three; Group Based Psycho-Education

Objectives

At the end of this sessions the trainee will be able to:

1. Describe psychoeducation interventions
2. Explain how to deliver psychoeducation interventions
3. Apply group psychoeducation interventions

Materials and preparation

- Flip chart
- Markers,

Time 2hrs minute

Activity /Discussion:

3. Ask: what is psycho-education and group education?
4. Listen to their responses and then share the following:

Group psychoeducation

Psychoeducation is a professionally delivered treatment modality that integrates and synergizes psychotherapeutic and educational interventions (Dixon, 1999; Marsh, 1992). The patient/client and/or family are considered partners with the provider in treatment, on the premise that the more knowledgeable the care recipients and informal caregivers are, the more positive health-related outcomes will be for all. To prepare participants for this partnership, psychoeducational techniques are used to help remove barriers to comprehending and digesting complex and

emotionally loaded information and to develop strategies to use the information in a proactive fashion.

Psychoeducation can be practiced one-on-one, group practice models set the stage for within-group dialogue, social learning, expansion of support and cooperation, the potential for group reinforcement of positive change, and network building (Penninx et al., 1999). They reduce isolation and serve as a forum for both recognizing and normalizing experience and response patterns among participants, as well as holding professionals accountable for high standards of service. Cognitive-behavioral techniques such as problem solving and role-play enhance the presentation of didactic material by allowing people to rehearse and review new information and skills in a safe setting. These can be amplified through specific attention to the development of stress management and other coping techniques (Anderson et al., 1986; McFarlane, 2002).

Element of psychoeducation's

- Briefing the client about psychosocial problems—in this case perinatal depression
- Stress management /prevention methods
- Social support as prevention methods of PPD
- Involve family as treatment partner

Goals of psycho-education's

- Ensuring client and their relatives' attainment of basic competency
- Facilitating an informed and self-responsible handling of the problem
- Deepening the client role as an expert
- Co-therapist -strengthening the role of relatives
- Optimal combination of professional therapeutic methods and empowerment
- Improving insight into illness and improving of compliance
- Promoting/supporting healthy life styles components
- Economizing informational and educational activities ETC.

Benefits of Psycho-educations

- Every person will feel more relaxed and in control of their condition if they have a greater level of understanding

- People who are educated about their condition are more likely to actively participate in their self - management and health promotion

Effective psychoeducation

- Education should interactive
- Use multiple teaching aids
- Connote consumer as the expert
- Elicit relative experience and understanding
- Avoid conflict and confrontation
- Education is a long -term process
- Evaluate understanding
- Review materials as often as possible

Group Psychoeducation's

- Group psychoeducation can be very effective for a number of reasons
- People feel that they the support of the group as well as the feeling they are not alone which decrease's the amount of stress in the situations
- Majority of common questions will be dealt with predominantly through discussion
- In a group situation experiences are shared with each other's. This way everyone gains a perspective of how other people deal with their situations which adds to the overall learning experience

Session Four; - Prevention Methods

Objective

To increase mother's ability to cope with stress in a healthy manner

Specific Objectives:

- Make mothers understand stress

- Provide mothers with essential knowledge on the possible causes and consequences of stress
- Help mothers to know the meaning and functions of coping
- Help mothers to clearly differentiate healthy and unhealthy way of coping stress
- Train mothers with the basic skills of developing healthy coping mechanisms

Materials and preparation

• Flip chart

• Markers

Time 60-90 minute

Activity /Discussion:

Brainstorming

Ask: what is stress?

- Have you experienced any stress?
- What was the reason?
- How do you overcome it?
- ➢ Listen to their responses and then share the following

Core issue about stress: Definition, Types and Sources of Stress, Negative Consequences

Definition

- Stress is a positive reaction we have as human beings which helps us to survive. It is an automatic response to everyday stimulus which if we were not alert to could cause us harm.
- Stress is also defined as pressure or tension felt by the individual. **Stress refers to physical, mental or emotional strain or tension caused** by overworking the mind, body and heart. **Lazarus (1986**

Types of Stress

Following are descriptions of the three types of stress that The National Scientific Council on the Developing Child has identified based on available research:

1. **Positive stress** results from adverse experiences that are **short-lived**.

Mothers may encounter positive stress when they attend a health facility, labor. This type of stress causes minor physiological changes including an increase in heart rate and changes in hormone levels.

With the support of caring partner/ friend or health care provider, mother can learn how to manage and overcome positive stress. This type of stress is considered normal and coping with it is an important part of the development process.

Brain storming

What are positive stresses prevalent during pregnancy and post-partum period?

Why you consider them as Positive?

b. Tolerable stress refers to adverse experiences that are **more intense but still relatively short-lived.**

Examples include the **death of a loved one**, **a natural disaster**, **a frightening accident**, and **family disruptions such as separation or divorce**.

If a mother has the support of friends, health care providers or responsible organizations, tolerable stress can usually be overcome. In many cases, tolerable stress can become positive stress.

However, if the mother lacks adequate support, tolerable stress can become toxic and lead to long-term negative bio psychosocial effects.

Brainstorming:

- What are tolerable stresses prevalent with your client?
- Why you consider them as tolerable?

c. Toxic stress results from intense adverse experiences that may be sustained **over a long period of time—weeks, months or even years.** This type of stress can disrupt the cognitive

potential, compromise the functioning of important biological systems, and lead to long-term health problems.

An example of toxic stress includes:

- **Abuse (intimate partner violence): -** Emotional, Physical, Sexual
- **Household Dysfunction: -** Economic problem, husband cheating, Household mental illness, divorce, Imprisoned household member.

3.2 Sources of Stress

Brain Storming

- What day-to-day hassles or events in the house hold have you experienced frequently?
- What kind of factors make you stressful?
- Where does stress come from, and what are its sources?

To answer this question, we will examine sources that arise within the person, in the family, and in the community and society.

The sources of stress may change as people develop, but the condition of stress can occur at any time throughout life.

There are a number of major events that occur in our lives which can be appraised as stressful.

Situations that are considered stress-provoking are known as stressors. Stressors are an internal or external stimulus event that places demand on organism for some kind of adaptive response.

What events are appraised as stressful?

1. Salient events
2. Overload
3. Ambiguous events
4. uncontrollable

Sources of stress can be

a) **Within the person**

Sometimes the source of stress is within the person. Source of stress within the person may include the followings: -

- **Different Kinds of Change like:**
  - Illness:
  - Age: Why is the person's age important?
  - **Type A syndrome -** complex pattern angry, competitive, and perfectionist behavior in response to stress. Perfectionism (placing high expectations on themselves and allowing no room for failure)
- **Opposing motivational forces (conflicting interest):**

Approach/approach conflict: arises when we are attracted toward two appealing goals that are incompatible. **Example:** Spending money to help family and spending for house hold problems.

Avoidance/avoidance conflict: occurs when we are faced with a choice between two undesirable situations. **Example:** avoiding loneliness and disliking joining friends/ neighbors who engage in bad habit who are not acceptable by your partner.

Approach/avoidance conflict: arises when we see attractive and unattractive features in a single goal or situation. This type of conflict can be stressful and difficult to resolve. Another example can be a mother interested to be with his partner but partner have cheating on her or have bad habit.

In general, people are likely to find conflict stressful when the choices involve many features, when opposing motivational forces have fairly equal strength, and when the "wrong" choice can lead to very negative and permanent consequences.

In addition to these stresses can be caused by:

- Doing more work than our mind, body or heart can bear or working overtime;
- Doing things, we do not enjoy doing;
- Doing routine work that gets boring with time;
- Working with people who are fading;
- Working to please others inadequate free time;
- Suppressing emotions like anger, sadness, affection etc.

b) **Sources from the family**

Family instability, financial pressure (dealing with the financial pressures which result from an extended period of work), death, illness and high family expectations can be potential sources which may put an individual to stress.

c) **Source from the environment**

- New environment and new friends/ neighbors: its common that first time mothers find themselves facing some amount of stress immediately after giving birth.
- Ambiguity—a lack of clarity in a situation— not clearly understanding medical conditions
- Preparing for labor
- Lack of health care provider/partner contact: health care provider involvement was an important ingredient in academic. Mothers need a deeper personal relationship with their health care provider. Yet the need for individual support and affirmation often goes unmet due to various factors.
- Excessive workload, inadequate free time, and little opportunity to influence the environment.

Case 1: There is a woman who is just pregnant for the first time. She went to a faraway place with her partner for the first time. She had begun to live with new neighbors and in-laws. She is lonely, has some pregnancy discomfort, and wants to get a visit from her neighbors or in-laws. She starts going out with her neighbors. They invited her to their home, but they did not understand her feelings.

3.3 Symptoms of stress

Discussion point

- What are the symptoms/indicators of stress?

We can identify **basic physical** and **cognitive** components that are good indicators of stress. Physiological components concern actual physical changes that occur. The following are several signs and symptoms:

- Decreased energy
- Sleep problems increased need for sleep or insomnia
- Weight loss or gain; decreased or increased appetite

- Accident proneness
- Increased susceptibility to illness
- Psychosomatic complaints headaches, migraines, ulcers or backaches
- Substance abuse -- excessive drinking or drug use
- Cardiopulmonary problems increased blood pressure or heart disease

Cognitive components also make up some of the primary manifestations of **stress and emotional fatigue**. Listed below are some of these factors:

- Depression: mood changes or cries easily
- Isolation: lack of desire to socialize, or simply isolates self from others either physically or emotionally
- Marital/family/roommate conflicts
- Cynicism
- Rigidity or passivity
- Aggression
- Mental illness
- Self-esteem problems: sens of despair, emptiness or sense of meaninglessness

Obviously, a person may not display all of these symptoms nor is this list comprehensive. One symptom may be enough to necessitate personal lifestyle change.

3.4 Possible Negative Consequences

Discussion point

- Identify the possible negative effects of stress and share experience with each other.

Stress may result in various negative consequences; some of them are the followings:

1) **Work potential and Quality of work affected***:* The quality of work produced in the course of a graduate career can be severely affected by poor coping strategies. Stress may produce a focus on short term completion versus long term learning. For instance, a fatigued mother may cram for a child care or parenting rather than master the activity, or she irritate, instead of effectively caring her

child. The result is that her work suffers, and she will not get the maximum value from her mothering.

2) **Family plans paused*:*** Without change the mother may yield to overwhelming fatigue which may lead to the stop her productive plans. Dreams of having healthy family, earning money and loving partner are shattered. Despite investment of years in marriage the grad will fall short of her perceived goals.

3) **Interpersonal relationships stunted***:* a mother may withdraw herself from social contact and loneliness may result. Many pressured mothers attempt to minimize other interests and limit their efforts to what is required within their marriage this causes an unhealthy isolation.

4) **Variety intellectual (cognitive) functions may be adversely affected** - attention, memory, decision making.

5) Easily fatigued (tired)
6) Restlessness and sleeplessness
7) Anger and Depression
8) Future career jeopardized (risked)

Stress Coping Mechanism

Case study

Assume you have rich neighbor in your village but you spend time going here and there with no purpose and no work. Considering your and their own achievement, your friends in one way or another told you that you don't have the capacity to perform as they do and you get into stressful situation. If you were in the same situation how do you react/cope to stress?

Discussion points

- What is coping?
- How you acted in response to stress?
- What you did to make yourself feel better?

Since everyone has a unique response to stress, there is no "one size fits all" solution to managing it. No single method works for everyone or in every situation, so experiment with different techniques and strategies is the better way for designing an effective coping mechanism. Generally coping is effective when it focusses on what makes you feel stressed and what makes you feel calm and in control.

Scenario 1:

W/r, Jamila is 7 months pregnant. While her gestational age increases, she experienced tension having due lack of relative who will be with her at health facility during labor. After a while she asked her old friend to be with her when she go to hospital for delivery. Finally, her old friend become volunteer and gave birth a healthy baby at hospital.

Scenario 2: **W/r** Nesra is 7 months pregnant. While her gestational age increases, she experienced tension having due lack of relative who will be with her at health facility during labor. So, she made a decision to not to ask anybody for help and give birth at home. Unfortunately, during labor, she bleeds much and she lost her child.

Discussion points

1) What method do the mothers use to cope with stress?
2) What was their focus in coping process?
3) What kind of coping method would you use if you were in similar position?
4) What do you think will be the consequence?

Coping is the process by which people try to manage the perceived discrepancy between the demands and resources they appraise in a stressful situation. Since people engage in coping in an effort to neutralize or reduce stress, coping activities are geared toward decreasing the person's appraisal of or concern for this discrepancy.

3.5.1 Functions and Methods of Coping

According to Richard Lazarus (1986) and his colleagues, coping can serve two main functions.

a. It can alter the problem causing the stress or

b. It can regulate the emotional response to the problem.

Coping can be grouped in to two groups based on the focus the coping process targets:

1) **Emotion-focused coping** is aimed at controlling the emotional response to the stressful situation. People can regulate their emotional responses through **behavioral** and **cognitive** approaches. Examples of behavioral approaches include using alcohol or drugs, seeking emotional social support from friends or relatives, and engaging in activities, such as sports or watching TV, Going to religious place/praying, that distract one's attention from the problem. **Cognitive approaches** involve how people think about the stressful situation.

2) **Problem-focused coping** is aimed at reducing the demands of the stressful situation or expanding the resources to deal with it. Everyday life provides many examples of problem-focused coping, including quitting stressful peer relationship, planning a new schedule for work (and sticking to it), seeking medical or psychological treatment, and learning new skills. People tend to use problem-focused approaches when they believe their resources or the demands of the situation are changeable.

In addition to the above classification from the point of an individual well-being, coping mechanisms can be divided in to two:

1. Healthy and
2. Unhealthy way of coping with stress

Discussion points

- What does healthy and unhealthy way of coping with stress?
- List the healthy and unhealthy ways of coping with stress?

These coping strategies may temporarily reduce stress, but they cause more damage in the long run:

**Taking out your stress on others (lashing out, angry outbursts, physical violence)**<br>**Overeating or under eating**<br>**Withdrawing from friends, family, and activities**<br>**Drinking too much or smoking**	**Using pills or drugs to relax**<br>**Sleeping too much**<br>**Procrastinating(postponing)**<br>**Filling up every minute of the day to avoid facing problems**

Healthy ways of coping with stress

Dealing with Stressful Situations: The Four A's

**Change the situation:**	**Change your reaction:**
Avoid the stressor	Adapt to the stressor
Alter the stressor	Accept the stressor

Avoid unnecessary stress

Not all stress can be avoided, and it's not healthy to avoid a situation that needs to be addressed. You may be surprised, however, by the number of stressors in your life that you can eliminate.

Learn how to say "no" – Know your limits and stick to them. Whether in your personal or professional life, refuse to accept added responsibilities when you're close to reaching them. Taking on more than you can handle is a surefire recipe for stress.

Avoid people who stress you out – If someone consistently causes stress in your life and you can't turn the relationship around, limit the amount of time you spend with that person or end the relationship entirely.

Take control of your environment –avoid places that may causes you feel stress.

Avoid hot-button topics – If you get upset over competitions about house hold economy, marriage or work, cross them off your conversation list. If you repeatedly argue about the same subject with the same people, stop bringing it up or excuse yourself when it's the topic of discussion.

Pare down your to-do list – Analyze your schedule, responsibilities, and daily tasks. If you've got too much on your plate, distinguish between the "should" and the "musts." Drop tasks that aren't truly necessary to the bottom of the list or eliminate them entirely.

Alter the situation

If you can't avoid a stressful situation, try to alter it. Figure out what you can do to change things so the problem doesn't present itself in the future. Often, this involves changing the way you communicate and operate in your daily life.

Express your feelings instead of bottling them up: If something or someone is bothering you, communicate your concerns in an open and respectful way. If you don't voice your feelings, resentment will build and the situation will likely remain the same.

Be willing to compromise: When you ask someone to change their behavior, be willing to do the same. If you both are willing to bend at least a little, you'll have a good chance of finding a happy middle ground.

Be more assertive: Don't take a backseat in your own life. Deal with problems head on, doing your best to anticipate and prevent them. If you've got an exam to study for and your chatty roommate just got home, say up front that it is not the time to talk.

Manage your time better: Poor time management can cause a lot of stress. When you're stretched too thin and running behind, it's hard to stay calm and focused. But if you plan ahead and make sure you don't overextend yourself; you can alter the amount of stress you're under.

Adapt to the stressor

If you can't change the stressor, change yourself. You can adapt to stressful situations and regain your sense of control by changing your expectations and attitude.

Reframe problems: Try to view stressful situations from a more positive perspective. Rather than fuming about a traffic jam, look at it as an opportunity to pause and regroup, listen to your favorite radio station, or enjoy some alone time.

Look at the big picture: Take perspective of the stressful situation. Ask yourself how important it will be in the long run. Will it matter in a month? A year? Is it really worth getting upset over? If the answer is no, focus your time and energy elsewhere.

Adjust your standards: Perfectionism is a major source of avoidable stress. Stop setting yourself up for failure by demanding perfection. Set reasonable standards for yourself and others, and learn to be okay with "good enough."

Focus on the positive: When stress is getting you down, take a moment to reflect on all the things you appreciate in your life, including your own positive qualities and gifts. This simple strategy can help you keep things in perspective.

Adjusting Your Attitude: How you think can have a profound effect on your emotional and physical well-being. Each time you think a negative thought about yourself, your body reacts as if it were in the throes of a tension-filled situation. If you see good things about yourself, you are more likely to feel good; the reverse is also true. Eliminate words such as "always," "never," "should," and "must." These are telltale marks of self-defeating thoughts.

Accept the things you can't change

Some sources of stress are unavoidable. You can't prevent or change stressors such as the death of a loved one, a serious illness, or a national recession. In such cases, the best way to cope with stress is to accept things as they are. Acceptance may be difficult, but in the long run, it's easier than railing against a situation you can't change.

Don't try to control the uncontrollable. Many things in life are beyond our control—particularly the behavior of other people. Rather than stressing out over them, focus on the things you can control such as the way you choose to react to problems.

Look for the upside. As the saying goes, “What doesn’t kill us makes us stronger.” When facing major challenges, try to look at them as opportunities for personal growth. If your own poor choices contributed to a stressful situation, reflect on them and learn from your mistakes.

Share your feelings. Talk to a trusted friend or make an appointment with a therapist. Expressing what you’re going through can be very cathartic, even if there’s nothing you can do to alter the stressful situation.

Learn to forgive. Accept the fact that we live in an imperfect world and that people make mistakes. Let go of anger and resentments. Free yourself from negative energy by forgiving and moving on.

Make time for fun and relaxation

Beyond a take-charge approach and a positive attitude, you can reduce stress in your life by nurturing yourself. If you regularly make time for fun and relaxation, you’ll be in a better place to handle life’s stressors when they inevitably come.

Healthy ways to relax and recharge

- Go for a walk.
- Spend time in nature.
- Consult a trusted friend.
- Sweat out tension with a good workout.
- Take a long bath.
- Savor a warm cup of coffee or tea.
- Get a massage.
- Curl up with a good book.
- Listen to music.
- Watch a comedy

Don’t get so caught up in the hustle and bustle of life that you forget to take care of your own needs. Nurturing yourself is a necessity, not a luxury.

- **Set aside relaxation time.** Include rest and relaxation in your daily schedule. Don’t allow other obligations to encroach. This is your time to take a break from all responsibilities and recharge your batteries.

- **Connect with others.** Spend time with positive people who enhance your life. A strong support system will buffer you from the negative effects of stress.

- **Do something you enjoy every day.** Make time for leisure activities that bring you joy, whether it be stargazing, playing the piano, or working on your bike.

Keep your sense of humor. This includes the ability to laugh at yourself. The act of laughing helps your body fight stress in a number of ways.

Learn the relaxation response

Adopt a healthy lifestyle

You can increase your resistance to stress by strengthening your physical health.

Exercise regularly: Physical activity plays a key role in reducing and preventing the effects of stress. Make time for at least 30 minutes of exercise, three times per week. Nothing beats aerobic exercise for releasing pent-up stress and tension.

Eat a healthy diet: Well-nourished bodies are better prepared to cope with stress, so be mindful of what you eat. Start your day right with breakfast, and keep your energy up and your mind clear with balanced, nutritious meals throughout the day.

Reduce caffeine and sugar: The temporary "highs" caffeine and sugar provide often end in with a crash in mood and energy. By reducing the amount of coffee, soft drinks, chocolate, and sugar snacks in your diet, you'll feel more relaxed and you'll sleep better.

- **Avoid alcohol, cigarettes, and drugs:** Self-medicating with alcohol or drugs may provide an easy escape from stress, but the relief is only temporary. Don't avoid or mask the issue at hand; deal with problems head on and with a clear mind.

Get enough sleep: Adequate sleep fuels your mind, as well as your body. Feeling tired will increase your stress because it may cause you to think irrationally.

Guidelines for being healthy

1) Try to understand context of your behavior by considering current situation and its relation to the past.
2) Give yourself and others only constructive criticism.
3) Compare your thoughts, feelings, and reactions to those of your peers so that you have a realistic standard.

4) Develop friendships and communicate regularly with friends, sharing your feelings, joys, and worries.
5) Develop a sense of balanced time perspective.
6) Take full credit for your successes and happiness, and share your positive feelings with other people.
7) When you feel you are losing control over your emotions, distance yourself from the situation physically by leaving it, role-playing another position, imagining the future to gain perspective on problem, and talking to sympathetic listener
8) Remember that failure and disappointment are sometimes blessings in disguise. Learn from them.
9) If you cannot help yourself, seek the counsel of trained specialist.
10) Cultivate healthy pleasures; give yourself permission to take time out, relax, meditate, get a massage, fly a kite, blow bubbles, and enjoy things you can do alone.

Session Five: Social Support and Partner Invitation

Objectives

- Describe social support ,its benefit for healthy coping and prevent PPD
- Discuss forms of social support and How can a person Improve the Social Support need?
- Advantage and disadvantage of social support

Materials and preparation

- Flip chart
- Markers

Time 3hrs minute

Activity /Discussion:

1. Ask: What is social support, its benefit for healthy coping and prevent PPD?
2. Listen to their responses and then share the following:

What is Social Support?

Social support is the physical and emotional comfort given to us by our family, friends, co-workers and others. It's knowing that we are part of a community of people who love and care for us, and value and think well of us. We all need people we can depend on during both the good times and the bad. Maintaining a healthy social support network is hard work and something that requires ongoing effort over time.

Forms of Social Support

Support can come in many different forms:

- **Emotional Support:** This is what people most often think of when they talk about social support. People are emotionally supportive when they tell us that they care about us and think well of us. For example, if you separated from your partner or lost your job, a close friend might call every day for the first few weeks afterwards just to see how you are doing and to let you know that he or she cares.
- **Practical Help:** People who care about us give us practical help such as gifts of money or food, assistance with cooking, child care, or help moving house. This kind of support helps us complete the basic tasks of day-to-day life.
- **Sharing Points of View:** Another way for people to help is to offer their opinion about how they view a particular situation, or how they would choose to handle it. In sharing points of view, we can develop a better understanding of our situation and the best way to handle it. For example, if you tell a friend about difficulties you are having with your teenage son, she may offer a point of view you hadn't considered, and this may help you to better address the situation with your child.
- **Sharing Information:** It can be very helpful when family, friends or even experts give us factual information about a particular stressful event. For example, a friend who recently married might provide information about the cost of various components of their wedding, or a cancer survivor might provide information about what to expect from different types of cancer treatment.

Getting Your Support Needs Met

Many of the people who are a part of our lives can provide social support. These can include our parents, spouse or partner, children, siblings, other family members, friends, co-workers, neighbors, health professionals and sometimes even strangers. We are unlikely to have all of our support needs met by just one person. Also, different people may be able to provide different types of support (e.g., our mother may be great at helping with child care, and our best friend might give great advice).

In general, the best support comes from the people we are closest to. Research has shown that receiving support from people we have close emotional ties to does more for our emotional and physical health than support provided by people we are not particularly close to. For example, having close friends listen and care for you during a stressful time will likely do more for you than receiving the same support from someone whom you don't know very well.

The Pros and Cons of Support

The Pros

Research tells us that it is important to have at least one close friend. Having many close friends may not do us any better than having only two or three close friends. Research also tells us that social support does the best job of protecting us from the effects of stress when we believe that emotional support is easy to come by, and we have at least one person we can confide in.

TheCons

On the other hand, unhappy or poor-quality relationships with other people have been shown to have a negative impact on mental health and well-being. Conflictual, distressing relationships may do us more psychological harm than positive social relationships can do us good. For example, compared with people who are single, separated or divorced, people who are married and can talk with their partners are less likely to have problems with depression. However, people who are married but cannot talk with their spouse are at much greater risk of clinical depression compared with others. It may be that poor-quality relationships impair our ability to cope with stress much more than good quality relationships protect us. The greatest benefit of social support may come from the protection from unhealthy relationships that it provides.

How Do I Improve My Social Support Network?

1. **Reactivate social networks**» Identify the person's prior social activities that, if reinitiated, would have the potential for providing direct or indirect psychosocial support (e.g. family gatherings, outings with friends, visiting neighbors, social activities at work sites, sports, community activities). » Build on the person's strengths and abilities and actively encourage to resume prior social activities as far as is possible.
2. **Don't be afraid to take social risks:** Making new friends and acquaintances means that you will have to take some risks. You will need to seek out and introduce yourself to new people. Ask a friend to help you if this is hard for you (e.g., come to a hospital or clinic with you). If you have always been shy, and this has got in the way of you finding the support you need, consider gaining the help of a professional. Of course, parties and events aren't the only social venues at which to meet people; informal gatherings/like edir, community centres/religious place, mother support groups, volunteer positions, schools and workplaces are also common places to meet people.
3. **Get more from the support you have:** While being careful not to overpower support providers, ask for what you need from others and be as specific as possible in your requests. It's a mistake to think that people will automatically know what you need - you will have to tell them.
4. **Ask for help:** Ask the people you know to help you broaden the network you have. If you have recently become single, ask your friends to introduce you to other single people your age. If you have recently come out, ask people you know who have gay friends to introduce you to them.
5. **Make a plan:** Figure out what kind of new support you need and brainstorm different ways you might be able to find it. Link to problem solving module once completed
6. **Create new opportunities:** Attend a religious activity and others. To meet new people, you will have to step outside your usual activities and lifestyle. If you just keep on doing what you always do, hoping to meet new people, you probably will fail.
7. **Let go of unhealthy ties:** This can be very difficult. Walking away from any relationship is painful. This is no less true because the relationship is doing you harm. But sometimes this is what we need to do. If all of your friends are involved in activities you want to avoid (e.g., using recreational drugs or shoplifting), you will need to let go of these friendships or

risk getting back into your old habits. Use your judgement - sometimes we can simply spend less time with certain people (e.g., friends who don't like to exercise or meet new people) without abandoning the friendship altogether.

8. **Protect your marriage:** We know that a good marriage offers protection from depression and a bad marriage makes us vulnerable. If your marriage is faltering, do what you can to improve it. Don't be afraid to seek professional help when you need it.
9. **Be a joiner:** Sometimes the best way to find the support you need is through a support group. If you need support for a highly specific problem (e.g., raising a child with Downs) you may only be able to find this support through a formal group setting.
10. **Be patient:** While very much worth the effort, making new friends is time-consuming. Recognize that you may need to meet many new people in order to make just one new friend. Building intimacy also takes time. It may be several months from the time that you meet someone before you feel really close to them and that you can count on their support.
11. **Avoid negative relationships:** We know that negative, conflictual relationships are hard on our emotional health. The negative aspects may be obvious (e.g., abuse) but other times they can be more subtle (e.g., excessive dependence or over-controlling). Sometimes it may be the other person's behavior that is the primary problem but more often we are also involved in some way - even if it is just the unhealthy ways we are responding to the negative person in our life. As much as possible, avoid long-term relationships that are more negative than positive. Sometimes this can be hard - especially when these relationships are with family members. In this case, try to limit the amount of contact with these people (or buffer that contact with other helpful supporters), and avoid relying on them for support.

Tips for Healthy Relationships

Healthy relationships have been shown to increase our happiness, improve health and reduce stress. Studies show that people with healthy relationships have more happiness and less stress. There are basic ways to make relationships healthy, even though each relationship is different. These tips apply to all kinds of relationships: friendships, work and family relationships, and romantic partnerships.

1. **Keep expectations realistic.** No one can be everything we might want them to be. Healthy relationships mean accepting people as they are and not trying to change them.

2. **Talk with each other.** It can't be said enough: communication is essential to healthy relationships.

    - Take the time. Really be there.
    - Genuinely listen. Do not interrupt or plan what you're going to say next. Try to fully understand their perspective.
    - Ask questions. Show you are interested. Ask about their experiences, feelings, opinions, and interests.
    - Share information. Studies show that sharing information helps relationships begin. Let people know who you are, but don't overwhelm with too much personal information too soon.

3. **Be flexible.** It is natural to feel uneasy about changes. Healthy relationships allow for change and growth.

1. **Take care of yourself, too.** Healthy relationships are mutual, with room for both people's needs.

2. **Be dependable.** If you make plans with someone, follow through. If you take on a responsibility, complete it. Healthy relationships are trustworthy.

3. **Fight fair.** Most relationships have some conflict. It only means you disagree about something; it does not have to mean you don't like each other.

    - Cool down before talking. The conversation will be more productive if you have it when your emotions have cooled off a little, so you don't say something you may regret later.
    - Use "I statement." Share how you feel and what you want without assigning blame or motives. E.g. "When you don't call me, I start to feel like you don't care about me" vs. "You never call me when you're away. I guess I'm the only one who cares about this relationship." Similarly use this technique for any help needed at this time.

- Keep your language clear and specific. Try to factually describe behavior that you are upset with, avoiding criticism and judgment. Attack the problem, not the person.

- Focus on the current issue. The conversation is likely to get bogged down if you pile on everything that bothers you. Avoid using “always” and “never” language and address one issue at a time.

- Take responsibility for mistakes. Apologize if you have done something wrong; it goes a long way toward setting things right again.

- Recognize some problems are not easily solved. Not all differences or difficulties can be resolved. You are different people, and your values, beliefs, habits, and personality may not always be in alignment. Communication goes a long way toward helping you understand each other and address concerns, but some things are deeply rooted and may not change significantly. It is important to figure out for yourself what you can accept, or when a relationship is no longer healthy for you.

4. **Be affirming.** According to relationship researcher John Gottman, happy couples have a ratio of 5 positive interactions or feelings for every 1 negative interaction or feeling. Express warmth and affection!

5. **Keep your life balanced.** Other people help make our lives satisfying but they cannot meet every need. Find what interests you and become involved. Healthy relationships have room for outside activities.

6. **It’s a process.** It might look like everyone on campus is confident and connected, but most people share concerns about fitting in and getting along with others. It takes time to meet people and get to know them. Healthy relationships can be learned and practiced, and keep getting better.

7. **Be yourself!** It’s much easier and more fun to be authentic than to pretend to be something or someone else. Healthy relationships are made of real people.

Partner invitation and discussion

Define PPD, its risk factors, the potential impact on the mother, pregnancy outcome, and long-term effects on the baby and family as a whole. The need for family help for healthy coping and prevention of PPD should be discussed. Finally, the area of support (Emotional (expressions of empathy, love, trust, and care), Instrumental (Tangible aid), Informational (Advice, and suggestions), and Appraisal were discussed by midwives.

Annexes

Training evaluation form

<table>
<tr><td colspan="5">Please answer the questions as honestly as you can to help improve future trainings.</td></tr>
<tr><td>S.n</td><td></td><td>Good</td><td>Fair</td><td>Poor</td></tr>
<tr><td></td><td>I would rate this training overall as</td><td></td><td></td><td></td></tr>
<tr><td></td><td>The content was</td><td></td><td></td><td></td></tr>
<tr><td></td><td>The amount of information was</td><td></td><td></td><td></td></tr>
<tr><td></td><td>Materials and visual aids were</td><td></td><td></td><td></td></tr>
<tr><td></td><td>Trainer facilitation was</td><td></td><td></td><td></td></tr>
<tr><td></td><td>The practice exercises were</td><td></td><td></td><td></td></tr>
<tr><td></td><td colspan="4">The length of the training was (please circle)
1. Too long
2. Too short
3. Just right
What could have made this training better?

Should anything be left out in future training?

List one thing you will do differently after participating in this training.

Comments:---</td></tr>
</table>

Pre-test/Post-test evaluation

S/R	Pre-test/post-test evaluation	Yes	No
01	Feeling unusually sad and teary may be a symptom of postpartum depression.	1	2
02	Sleeping too much or too little may be a sign of postpartum depression.		
03	Eating too much or losing interest in food may be a sign of postpartum depression.		
04	Loss of interest or pleasure in most things may be a symptom of postpartum depression.		
05	Postpartum depression affects person's memory and concentration.		
06	Symptoms and signs of postpartum depression last for a period of at least 2 weeks.		
07	Postpartum depression might be caused by a genetic or inherited problem?		
08	Postpartum depression might be caused by stressful circumstances in the life (such as the death of a loved one or divorce)?		
09	Postpartum depression might be caused by a lack of social support such as intimate partner support?		
10	Postpartum depression might be caused by a previous history of depression? .		
11	Postpartum depression might be caused by a hormonal imbalance?		
12	Physical activity is effective for the prevention or management of postpartum depression.		
13	Seeking help with tasks like infant care and house hold chores from intimate partner and family members is helpful for the prevention or management of postpartum depression		
14	Religious practices, prayer and going to holy shrine are helpful for the prevention or management of postpartum depression		
15	Having a balanced diet is helpful for the prevention or management of postpartum depression		
16	Good sleep is helpful in prevention or management of postpartum depression.		
17	Although there are clinics for with postpartum depression, I would not have		

	much faith in them.		
18	Treatment for postpartum depression, provided by a mental health professional, can be effective		
19	Psychotherapy (for example, counseling) can be effective in treating postpartum depression		
20	Antidepressants are addictive		
21	Antidepressants cause brain damage		
22	I would rather live with postpartum depression than go through the ordeal of getting psychiatric treatment		
23	Most women who have postpartum depression are violent		
24	It is best to avoid women with postpartum depression so that you don't develop this problem		
25	If I had postpartum depression, I would not tell anyone		
26	I am afraid of what my family and/or friends might think of me for attending psychology and/or psychiatry appointments		
27	I know where to seek information about postpartum depression		
28	I know how to use various sources to seek information about postpartum depression		
29	I can appraise the accuracy of information about postpartum depression on the radio and television		
30	I can appraise the accuracy of information about postpartum depression on the Internet		
31	I can appraise the accuracy of advices about postpartum depression which given me by friends and family members		

Reference

Dadi, A.F., et al., *Global burden of antenatal depression and its association with adverse birth outcomes: an umbrella review.* BMC Public Health, 2020. **20**(1): p. 173.

Hahn-Holbrook, J., T. Cornwell-Hinrichs, and I. Anaya, *Economic and Health Predictors of National Postpartum Depression Prevalence: A Systematic Review, Meta-analysis, and Meta-Regression of 291 Studies from 56 Countries.* Front Psychiatry, 2017. **8**: p. 248.

Fekadu Dadi, A., E.R. Miller, and L. Mwanri, *Antenatal depression and its association with adverse birth outcomes in low and middle-income countries: Step 4: Review practice against evidence-based audit criteria.* PLoS One, 2020. **15**(1): p. e0227323.

Reference

O'Hara, M.W. and K.L. Wisner, *Perinatal mental illness: definition, description and aetiology.* Best Pract Res Clin Obstet Gynaecol, 2014. **28**(1): p. 3-12.

Fekadu Dadi, A., E.R. Miller, and L. Mwanri, *Antenatal depression and its association with adverse birth outcomes in low and middle-income countries: Step 4: Review practice against evidence-based audit criteria.* PLoS One, 2020. **15**(1): p. e0227323.

Ongeri, Lukens EP, Mcfarlane WR. Psychoeducation As Evidence-Based Practice: Considerations For Practice, Research, And Policy. Brief Treatment And Crisis Intervention. 2004;4(3):205-

Desta M, Memiah P, Kassie B, Ketema DB, Amha H, Getaneh T, Et Al. Postpartum Depression And Its Association With Intimate Partner Violence And Inadequate Social Support In Ethiopia: A Systematic Review And Meta-Analysis. Journal Of Affective Disorders. 2021;279:737-48. 45

National Institute Of Health And Care Excellence(192). Antenatal-And-Postnatal-Mental-Health-Clinical-Management-And-Service Guidance(2014).Wwwniceorguk/Guidance/Cg192.{Accessed December 17.2014}.

Johnson JE, Wiltsey-Stirman S, Sikorskii A, Miller T, King A, Blume JL, Et Al. Protocol For The ROSE Sustainment (ROSES) Study, A Sequential Multiple Assignment Randomized Trial To Determine The Minimum Necessary Intervention To Maintain A Postpartum Depression Prevention Program In Prenatal Clinics Serving Low-Income Women. Implement Sci. 2018;13(1):115.

O'Hara, M.W. and K.L. Wisner, *Perinatal mental illness: definition, description and aetiology.* Best Pract Res Clin Obstet Gynaecol, 2014. **28**(1): p. 3-12.
