# Supplementary material for: Effectiveness of group-based psycho-education on preventing postpartum depression among pregnant women by primary healthcare provider in primary healthcare institution: a cluster-randomized controlled trial
Source: Front Psychiatry. 2024 Sep 10;15:1433942. doi: 10.3389/fpsyt.2024.1433942 (PMC11420118; doi:10.3389/fpsyt.2024.1433942)
Supplement: Supplementary file 1 [file DataSheet1.zip › Supplementary Table 1-CONSORT checklist.pdf]

Table 1: CONSORT 2010 checklist of information to include when reporting a cluster randomised trial randomized

Section/Topic	Item No	Standard Checklist item	Extension for cluster designs	Page No *
Title and abstract				
	1a	Identification as a randomised trial in the title	Identification as a cluster randomised trial in the title	
	1b	Structured summary of trial design, methods, results, and conclusions (for specific guidance see CONSORT for abstracts)		
Introduction				
Background and objectives	2a	Scientific background and explanation of rationale	Rationale for using a cluster design	
	2b	Specific objectives or hypotheses	Whether objectives pertain to the cluster level, the individual participant level or both	
Methods				
Trial design	3a	Description of trial design (such as parallel, factorial) including allocation ratio	Definition of cluster and description of how the design features apply to the clusters	
	3b	Important changes to methods after trial commencement (such as eligibility criteria), with reasons		
Participants	4a	Eligibility criteria for participants	Eligibility criteria for clusters	
	4b	Settings and locations where the data were collected		
Interventions	5	The interventions for each group with sufficient details to allow replication, including how and when they were administered	Whether interventions pertain to the cluster level, the individual participant level, or both	
Outcomes	6a	Completely defined pre-specified primary and secondary outcome measures, including how and	Whether outcome measures pertain to the cluster level, the individual participant level or both	

		when they were assessed		
	6b	Any changes to trial outcomes after the trial commenced, with reasons		
Sample size	7a	How sample size was determined	Method of calculation, number of clusters(s) (and whether equal or unequal cluster sizes are assumed), cluster size, a coefficient of Intracluster correlation (ICC or *k*), and an indication of its uncertainty	
	7b	When applicable, explanation of any interim analyses and stopping guidelines		
Randomisation:				
Sequence generation	8a	Method used to generate the random allocation sequence		
	8b	Type of randomisation; details of any restriction (such as blocking and block size)	Details of stratification or matching if used	
Allocation concealment mechanism	9	Mechanism used to implement the random allocation sequence (such as sequentially numbered containers), describing any steps taken to conceal the sequence until interventions were assigned	Specification that allocation was based on clusters rather than individuals and whether allocation concealment (if any) was at the cluster level, the individual participant level or both	
Implementation	10	Who generated the random allocation sequence, who enrolled participants, and who assigned participants to interventions	Replace by 10a, 10b and 10c	
	10a		Who generated the random allocation sequence, who enrolled clusters, and who assigned clusters to interventions	
	10b		Mechanism by which individual participants were included in clusters for the purposes of the trial (such as complete	

			enumeration, random sampling)	
	10c		From whom consent was sought (representatives of the cluster, or individual cluster members, or both), and whether consent was sought before or after randomisation	
Blinding	11a	If done, who was blinded after assignment to interventions (for example, participants, care providers, those assessing outcomes) and how		
	11b	If relevant, a description of the similarity of interventions		
Statistical methods	12a	Statistical methods used to compare groups for primary and secondary outcomes	How clustering was taken into account	
	12b	Methods for additional analyses, such as subgroup analyses and adjusted analyses		
Results				
Participant flow (a diagram is strongly recommended)	13a	For each group, the numbers of participants who were randomly assigned, received intended treatment, and were analysed for the primary outcome	For each group, the numbers of clusters that were randomly assigned, received intended treatment, and were analysed for the primary outcome	
	13b	For each group, losses and exclusions after randomisation, together with reasons	For each group, losses and exclusions for both clusters and individual cluster members	
Recruitment	14a	Dates defining the periods of recruitment and follow-up		
	14b	Why the trial ended or was stopped		
Baseline data	15	A table showing baseline demographic and clinical	Baseline characteristics for the individual and cluster levels as	

		characteristics for each group	applicable for each group	
Numbers analysed	16	For each group, number of participants (denominator) included in each analysis and whether the analysis was by original assigned groups	For each group, number of clusters included in each analysis	
Outcomes and estimation	17a	For each primary and secondary outcome, results for each group, and the estimated effect size and its precision (such as 95% confidence interval)	Results at the individual or cluster level as applicable and a coefficient of Intracluster correlation (ICC or k) for each primary outcome	
	17b	For binary outcomes, presentation of both absolute and relative effect sizes is recommended		
Ancillary analyses	18	Results of any other analyses performed, including subgroup analyses and adjusted analyses, distinguishing pre-specified from exploratory		
Harms	19	All-important harms or unintended effects in each group (for specific guidance see CONSORT for harms[1])		
Discussion				
Limitations	20	Trial limitations, addressing sources of potential bias, imprecision, and, if relevant, multiplicity of analyses		
Generalisability	21	Generalisability (external validity, applicability) of the trial findings	Generalisability to clusters and/or individual participants (as relevant)	
Interpretation	22	Interpretation consistent with results, balancing benefits and harms, and considering other relevant evidence		
Other information				
Registration	23	Registration number and		

		name of trial registry		
Protocol	24	Where the full trial protocol can be accessed, if available		
Funding	25	Sources of funding and other support (such as supply of drugs), role of funders		

** Note: page numbers optional depending on journal requirements*
