# Supplementary material for: Effectiveness of group-based psycho-education on preventing postpartum depression among pregnant women by primary healthcare provider in primary healthcare institution: a cluster-randomized controlled trial
Source: Front Psychiatry. 2024 Sep 10;15:1433942. doi: 10.3389/fpsyt.2024.1433942 (PMC11420118; doi:10.3389/fpsyt.2024.1433942)
Supplement: Supplementary file 1 [file DataSheet1.zip › Supplementary Table 2-Description of the study variables.pdf]

Table 2. Description of the study variables

Variable	Description	Measurement
**Dependent variable**		
Postpartum depression	Mothers were assessed by the PHQ-9 tool during the 6-week postpartum period. It comprises 9 questions, each having scores of 0–3, with minimum and maximum scores of 0 and 27, respectively.	Score: (0–9) normal, (≥10) depressed (52-54).
**Individual level**		
Age	Age of the mother in completed years	A continuous variable and recoded into three categories: 15–24, 25–34, and ≥35
Education	The highest level of education the mother completed	Categorized into four groups: informal education, primary education (1–8), secondary education (9–12), and college and above
Marital status	Marital status of the mother	Categorized into three categories: married, in-relationship, and other (divorced, widowed, separated)
Job	The job of the mother	Categorized into four groups: housewife, private worker, government employee, and domestic worker
Estimated household income	The average monthly household earnings	A continuous variable and recoded into three categories: <3000, 3001–5000, and >5000
Chronic health history	Any chronic health condition the mother had	Coded as 'Yes' or 'No'
Gravidity	The number of times that a mother has been pregnant	Coded as 'Primipara' or 'Multipara'
Unwanted pregnancy (mother)	Mothers were asked if the pregnancy occurred when no children or no more children were desired (on her side)	Coded as 'Wanted' or 'Unwanted'
Complication during labor	The mother was asked if she faced complications during labor (like prolonged labor, obstructed labor, or operative delivery)	Coded as 'Yes' or 'No'
Exclusive breastfeeding	The mother was asked if she feeds her baby only breast milk, except for medications or vitamin and mineral supplements	Coded as 'Yes' or 'No'
Loneliness	Lack of rewarding social contact and/or relationships is not met.	Coded as 'Yes' or 'No'
Stressful life in the last 12 months	Major changes or losses in the last 12 months (e.g., death of a loved one,	Coded as 'Yes' or 'No'

	unemployment, bereavement, and migration)	
Literacy	Knowledge and beliefs about postpartum depression aid their recognition, management, or prevention. It comprises seven categories and 31 items: each item was rated on a 5-point Likert scale.	The median for overall postpartum depression literacy was computed and mothers who scored at and above the median were considered to have good PPD literacy and those who computed below the median were considered as poor PPD literacy (56).
History of mental illness	History of mental illness (e.g., eating disorder, psychosis, bipolar disorder, schizophrenia)	Coded as 'Yes' or 'No'
Self-esteem	Measured by the 10-item scale with a 4-point scale, ranging from 1 point (strongly disagree) to 4 points (strongly agree)	A continuous variable and computed. The mean for overall self-esteem was computed and mothers who scored above the mean were considered to have good self-esteem and those who computed below the mean were considered as poor self-esteem (70).
Coping strategy	Coping skill comprises 28 items with three dimensions of coping strategy: problem-focused, emotion-focused, and avoidance or dysfunctional coping.	The mean of the three dimensions of coping strategy was computed and mothers who scored at and above the mean were considered as having good coping skills, and those who computed below the mean were considered as having poor coping skills (57)
**Community level**		
Residence	The place where the respondent usually belongs	Coded as 'urban' and 'rural'
Interventions	Health centers/clusters were identified as intervention and control based on the intervention (group based on psycho-education) received	Coded as intervention = '1' and control = '0'
Social support	A functional social support questionnaire was used for the measurement of social support. It has 14 items with four categories: quantity of support, confidant support, affective support, and instrumental support.	After tool validation, the ordinal-rated response options were replaced with dichotomous (i.e., yes/no) variables. So, it was recoded as (yes = '1', no = '0'). In the end, the mean for overall social support was computed and mothers who scored above the mean were considered to have adequate social support and those who computed below the mean were considered to have

		inadequate social support (55)
Unwanted pregnancy (for partner)	The mother was asked if the current pregnancy occurred when no children or no more children were desired by the father of the baby	Coded as ‘Wanted’ or ‘Unwanted’
Emotionally supportive partner	Partner emotionally supportive or not was assessed by 1 item with a 5-point Likert scale	Recoded into ‘Yes’ (agree and strongly agree) or ‘No’ (neutral, disagree and strongly disagree)
Intimate partner violence	Twenty items are categorized into five forms: history of physical, sexual, psychological, economic violence, and controlling behavior	From the 20 items, if a mother experienced one type of violence, she considered it as if she had violence
Support from own mother	When the mother growing up, whether her mother was emotionally supportive or not was assessed by 1 item with a 5-point Likert scale	Recoded into ‘Yes’ (agree and strongly agree) or ‘No’ (neutral, disagree and strongly disagree)
Relationship with in-laws	Relationship with in-laws assessed by 1 item with a 5-point Likert scale	Recoded into ‘Yes’ (agree and strongly agree) or ‘No’ (neutral, disagree and strongly disagree)
Childhood abuse	Experience of emotional, physical, and sexual abuse when mother growing up	Coded as ‘Yes’ or ‘No’
Neonatal complications	Presence of neonatal complication (up to 28 days of age) (e.g., sepsis, asphyxia, early neonatal death)	Coded as ‘Yes’ or ‘No’
Child sleeping problem	Child sleeping problems or crying	Coded as ‘Yes’ or ‘No’
Birth outcome	Birth outcomes include normal-term birth, preterm birth, stillbirth, low birth weight, and congenital anomalies.	Coded as ‘Yes’ (normal term birth) or ‘No’ (preterm birth, stillbirth, low birth weight, and congenital anomalies)
